# Automatic ^13^C chemical shift reference correction for unassigned protein NMR spectra

**DOI:** 10.1007/s10858-018-0202-5

**Published:** 2018-08-10

**Authors:** Xi Chen, Andrey Smelter, Hunter N. B. Moseley

**Affiliations:** 1 Department of Molecular and Cellular Biochemistry, University of Kentucky, Lexington, KY 40356 USA; 2 Department of Statistics, University of Kentucky, Lexington, KY 40356 USA; 3 Markey Cancer Center, University of Kentucky, Lexington, KY 40356 USA; 4 Center for Environmental and Systems Biochemistry, University of Kentucky, Lexington, KY 40356 USA; 5 Institute for Biomedical Informatics, University of Kentucky, Lexington, KY 40356 USA

**Keywords:** Carbon chemical shift, Reference correction, Protein NMR, Statistical modeling

## Abstract

Poor chemical shift referencing, especially for ^13^C in protein Nuclear Magnetic Resonance (NMR) experiments, fundamentally limits and even prevents effective study of biomacromolecules via NMR, including protein structure determination and analysis of protein dynamics. To solve this problem, we constructed a Bayesian probabilistic framework that circumvents the limitations of previous reference correction methods that required protein resonance assignment and/or three-dimensional protein structure. Our algorithm named Bayesian Model Optimized Reference Correction (BaMORC) can detect and correct ^13^C chemical shift referencing errors before the protein resonance assignment step of analysis and without three-dimensional structure. By combining the BaMORC methodology with a new intra-peaklist grouping algorithm, we created a combined method called Unassigned BaMORC that utilizes only unassigned experimental peak lists and the amino acid sequence. Unassigned BaMORC kept all experimental three-dimensional HN(CO)CACB-type peak lists tested within ± 0.4 ppm of the correct ^13^C reference value. On a much larger unassigned chemical shift test set, the base method kept ^13^C chemical shift referencing errors to within ± 0.45 ppm at a 90% confidence interval. With chemical shift assignments, Assigned BaMORC can detect and correct ^13^C chemical shift referencing errors to within ± 0.22 at a 90% confidence interval. Therefore, Unassigned BaMORC can correct ^13^C chemical shift referencing errors when it will have the most impact, right before protein resonance assignment and other downstream analyses are started. After assignment, chemical shift reference correction can be further refined with Assigned BaMORC. These new methods will allow non-NMR experts to detect and correct ^13^C referencing error at critical early data analysis steps, lowering the bar of NMR expertise required for effective protein NMR analysis.

**Electronic supplementary material:**

The online version of this article (10.1007/s10858-018-0202-5) contains supplementary material, which is available to authorized users.

## Introduction

Nuclear magnetic resonance (NMR) is a highly versatile analytical technique for studying molecular configuration, conformation, and dynamics, especially of biomacromolecules such as proteins (Saitô 1986; Spera and Bax 1991; Wishart et al. 1991; Iwadate et al. 1999; Wishart and Case 2001; Neal et al. 2003; Mao et al. 2011; Serrano et al. 2012; Rosato et al. 2012). Several factors are fundamental to the utilization of NMR spectral data: resonance sensitivity, spectral precision, and spectral accuracy (De Dios et al. 1993; Vila et al. 2008). While various improvements in sample preparation (Wu et al. 2010; Akira et al. 2012), instrumentation (Yang and Bax 2010; Barette et al. 2011; Lange et al. 2012; Vernon et al. 2013), and pulse sequences (Meissner and Sørensen 2001; Khaneja et al. 2005) have greatly improved resonance sensitivity and spectral precision, spectral accuracy still depends on the same basic procedure: referencing chemical shifts to a designated chemical standard. Additionally, variance in chemical shifts can be caused by a variety of experimental factors, including pH, temperature, salts, organic solvent mixtures, and inaccurate referencing due to human error (Nowick et al. 2003; Ulrich et al. 2008). In protein NMR analyses, 4,4-dimethyl-4-silapentane-1-sulfonic acid (DSS) is the recommended internal standard for chemical shift referencing (Wishart et al. 1995; Markley et al. 1998). However, DSS has a negative charge at NMR-relevant pHs and can interact with positively charged residues of a protein of interest, broadening and altering its reference chemical shift values (Nowick et al. 2003). Additionally, temperature affects the reference chemical shift of DSS. Lack of experience or familiarity with chemical shift referencing and the factors that can affect referencing is a major contributor to chemical shift referencing inaccuracy. All downstream analyses and interpretations are affected by these inaccuracies in chemical shifts, including the assignment of resonances in biomacromolecules such as proteins. Moreover, these inaccuracies can outright prevent data analysis, especially with semiautomated data analysis tools, or propagate through data analysis, snowballing into interpretive errors about structure and dynamics. Since the structural and dynamic information contained in the chemical shift is subtle, even small chemical shifts errors due to inaccurate referencing may provide a distorted representation of the protein, especially if the chemical shifts are directly used in structure determination (Wu et al. 2010; Yang and; Bax 2010; Barette et al. 2011; Lange et al. 2012).

**Fig. 1:**
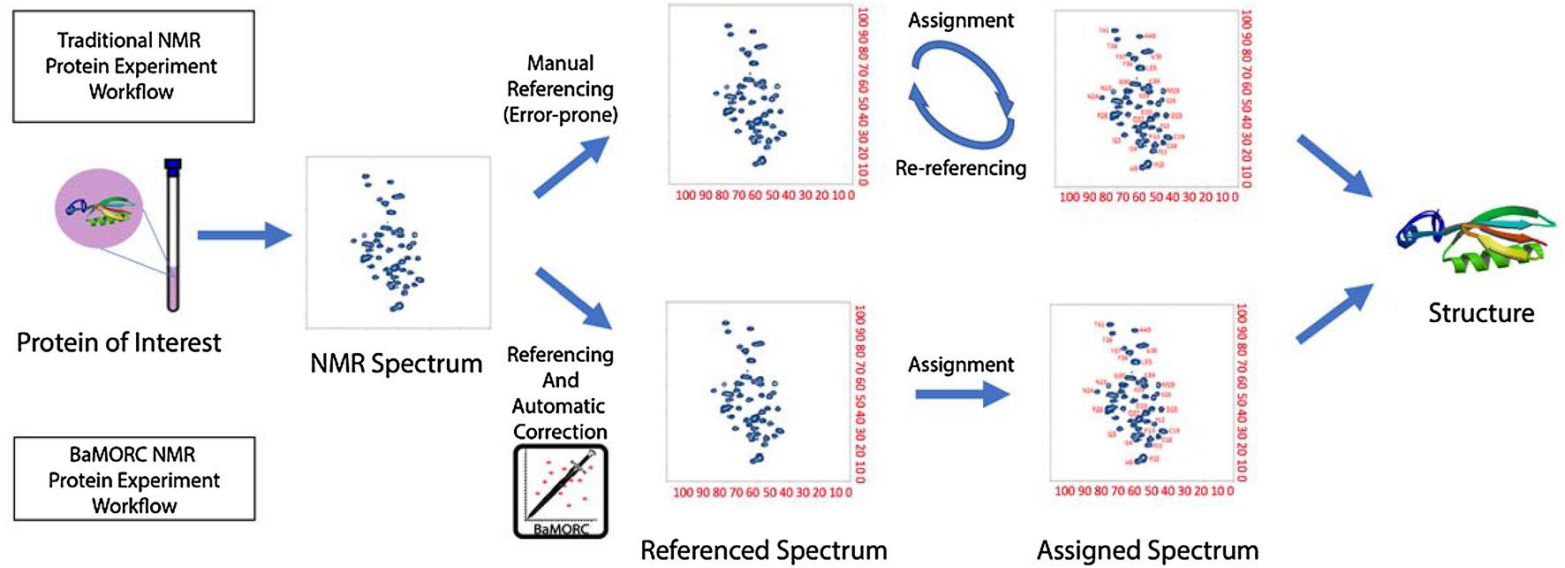
Overview of traditional and Unassigned BaMORC protein NMR referencing workflows. Top: The traditional workflow requires a manual referencing at step 2 to resolve the assignment initially, followed by refinement of referencing through a trial and error process. Bottom: The Unassigned BaMORC workflow allows referencing correction before assignment

Supplemental Table 2 shows several available programs used by the biomolecular NMR community for correcting referencing in ^1^H, ^13^C and ^15^N chemical shifts (Wishart 2011). In addition, there are a variety of tools for detecting protein resonance assignment errors, which can be due to bad referencing. These tools include but are not limited to AVS (Moseley et al. 2004), PANAV (Wang et al. 2010), CheckShift (Ginzinger et al. 2007; Wang et al. 2010), SHIFTX2 (Han et al. 2011) and VASCO (Rieping and Vranken 2010). Due to the complexity of manual procedures and various experimental factors, approximately 40% of the entries in the Biological Magnetic Resonance Bank (BMRB) have chemical shift accuracy problems (Wang et al. 2005; Ulrich et al. 2008). Unfortunately, current reference correction methods are heavily dependent on the availability of assigned protein chemical shifts or protein structure. One of the best examples is the SHIFTX program (Wang et al. 2005), which is used by the Re-referenced Protein Chemical shift Database (RefDB) (Zhang et al. 2003) to predict protein ^1^H, ^13^C and ^15^N chemical shifts from the X-ray or NMR coordinate data of previously assigned proteins to check and correct referencing using the companion program SHIFTCOR (Zhang et al. 2003). Another good example is the linear analysis of chemical shifts (LACS) method, which was developed by the National Magnetic Resonance Facility at Madison and the associated Biological Magnetic Resonance Bank (BMRB) and employs assigned chemical shifts to directly calculate a reference correction (Wang et al. 2005). However as shown in Fig. 1, this dependence on assigned shifts creates a vicious cycle between referencing and assignment in NMR spectra analysis: a correct chemical shift reference is required for good resonance assignment, and a good resonance assignment is needed to validate and correct chemical shift referencing. From a statistical analysis perspective, neither chemical shift referencing nor resonance assignment can be assessed independently of each other.

To address these issues in protein NMR, we have developed a new methodology referred to as Bayesian Model Optimized Reference Correction (BaMORC), which detects and corrects ^13^C chemical shift referencing errors using sets of C_α_ and C_β_ chemical shift pairs. BaMORC minimizes the difference between the known amino acid frequencies based on the protein sequence and the frequencies predicted using a set of mostly bivariate statistical models that are amino acid and secondary structure specific (Wishart et al. 1991) and are based on C_α_ and C_β_ chemical shift statistics. The minimization comes from the adjustment of the ^13^C chemical shift referencing. The statistical models integrate prior amino acid and chemical shift propensity information along with amino acid and secondary structure probabilities calculated using a chi-squared statistic based on C_α_ and C_β_ chemical shifts and refined chemical shift statistics derived from the RefDB. The refined expected values, variances, and covariances for C_α_ and C_β_ chemical shifts are derived from 1557 RefDB datasets of chemical shifts assigned using a variety of statistically driven data mining methods. Since RefDB only includes datasets from proteins with well-defined structure, BaMORC is likewise tuned to work with chemical shift datasets from proteins with well-defined structure. We integrated BaMORC with a new intra-peak list grouping algorithm (Smelter et al. 2017) developed in our laboratory to create a combined method, which we refer to as Unassigned BaMORC, that can correct ^13^C chemical shift referencing using unassigned three-dimensional HN(CO)CACB-type peak lists (Grzesiek and Bax 1992). Thus, ^13^C chemical shift referencing can be automatically analyzed and corrected before downstream analyses, including protein resonance assignment. Unassigned BaMORC generates a correction value, a file of re-referenced chemical shifts and a residual plot, which shows the optimization of the predicted amino acid frequencies and the point at which the best reference correction value occurs in the optimization. Furthermore, we have implemented an Assigned BaMORC method that can utilize assigned chemical shifts to improve reference correction.

## Results

### Deriving initial alpha carbon and beta carbon statistics from the RefDB

**Fig. 2:**
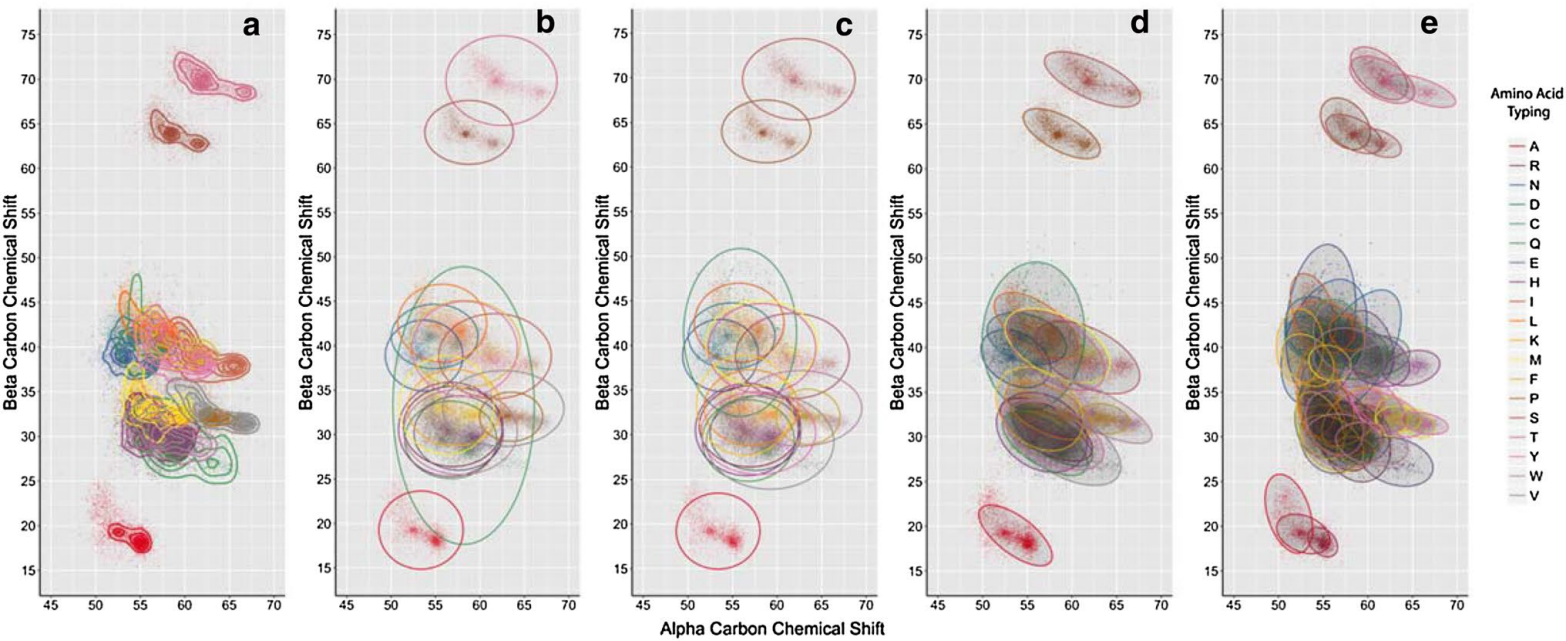
2D Distributions of alpha and beta carbon chemical shifts specific to amino acid and secondary structure types. **a** Real, i.e., true, bivariate distributions with density. **b** Statistically modeled distributions without covariance. **c** same as b but with oxidized and reduced cysteines represented as separate distributions. **d** Statistically modeled distributions with covariance. **e** Statistically modeled distributions with covariance for three secondary structure types

We downloaded all referenced BMRB entries and associated data from the RefDB on May 4th, 2015. Next, we extracted all relevant ^13^C chemical shift entries (datasets) as described in the Methods. Each dataset contains the protein sequence and the corresponding NMR chemical shifts. One point worth mentioning is that most of the datasets are not complete: i.e., there are fewer assigned residues than would be expected from the protein sequence. However, missing resonance assignments are common due to a myriad of experimental conditions, especially conformational flexibility in the protein structure that leads to intermediate chemical exchange. Using the secondary structure information accompanying the NMR chemical shift data provided by the RefDB, we associated residue-specific C_α_ and C_β_ chemical shifts and then sub-grouped them by amino acid and secondary structure type, as shown in Supplemental Fig. 1 for 19 of the 20 common amino acids (not including glycine) in proteins and for the secondary structure types helix, sheet, and coil. The univariate C_α_ and C_β_ chemical shift distributions are multimodal, with most of the modes being secondary structure specific (Spera and Bax 1991; Wishart et al. 1991). Next, we calculated the mean and standard deviation specific to the amino acid and secondary structure type and verified these statistics with the values provided by the RefDB. We then calculated the covariances between alpha and beta carbons. Figure 2 illustrates the overlapping alpha and beta carbon distributions for the 20 common amino acids minus glycine, and it demonstrates the reason why simple statistical models are inadequate without considering secondary structure, reduced/oxidized cysteines, and covariances. Figure 2a shows the distribution of all the RefDB data with contouring for the 19 common amino acids with both C_α_ and C_β_. Figure 2b shows these distributions represented with simple, independent bivariate models for each amino acid, as illustrated by ellipses centered on C_α_ and C_β_ chemical shift means, with the axes representing 2 standard deviations and providing approximately 95% coverage of the data. Figure 2c illustrates the same independent bivariate models, but with oxidized and reduced cysteines modeled separately. Figure 2d illustrates bivariate models with covariance. Figure 2e illustrates 60 bivariate models with covariance for the 19 common amino acids, sub-divided by secondary structure categories helix, sheet, and coil and with cysteine further divided into oxidized and reduced forms. These final 60 bivariate models match the observed distributions derived from RefDB data asymptotically and represent a key ingredient in the BaMORC methodology. The alpha and beta ^13^C chemical shift statistics used in these models are summarized in Supplemental Table 1.

### Separating bivariate distributions of alpha and beta carbons for oxidized and reduced cysteine residues

**Fig. 3:**
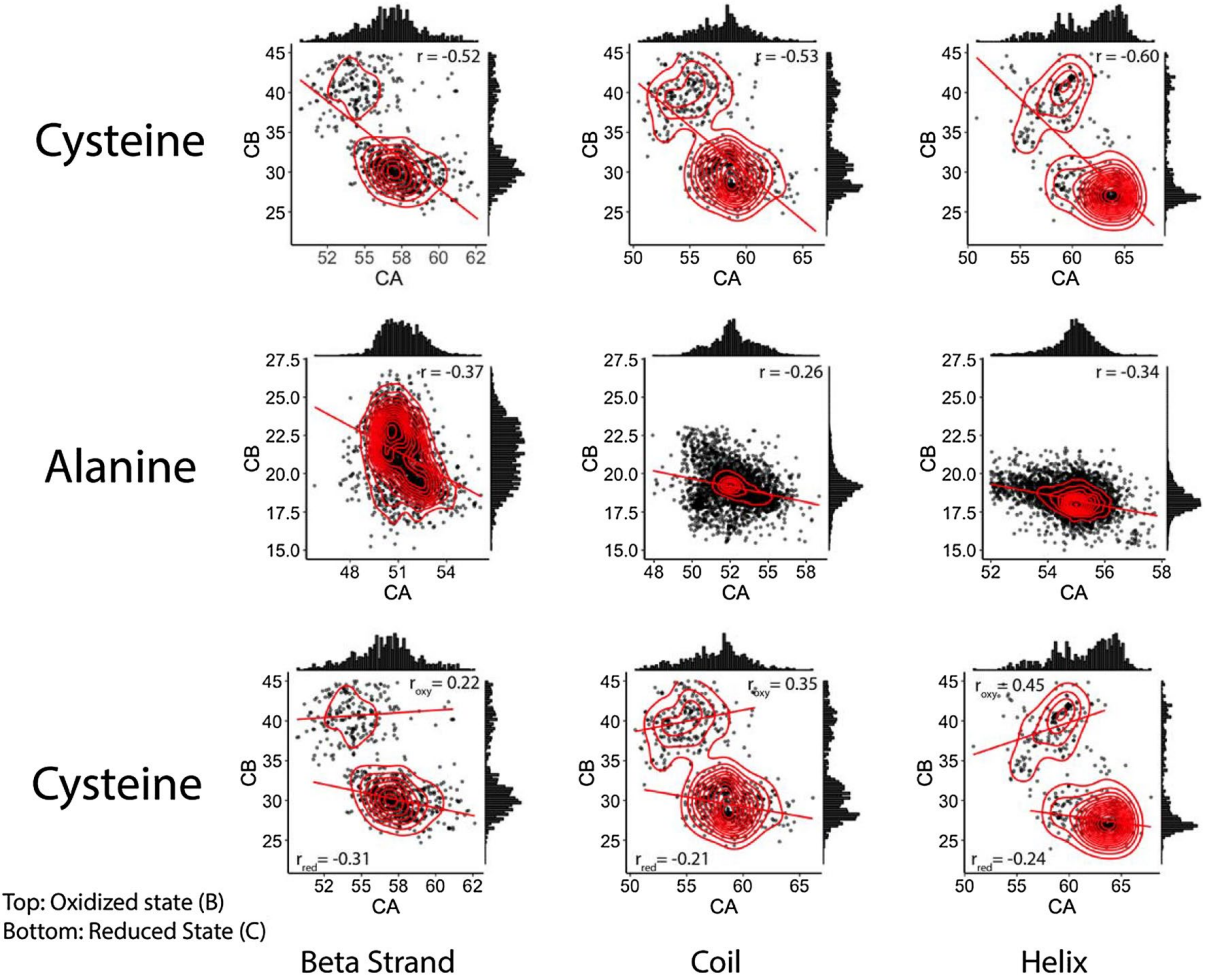
Top two panels: Amino acid distributions for alanine and cysteine, with corresponding correlation values. Top: Cysteine as a single population that appears as two. The correlation r values are − 0.52, − 0.53 and − 0.6 for beta strand, coil and helix secondary structure types. Bottom: Alanine shows a single mode and the correlation r values are − 0.37, − 0.26 and − 0.34 for beta strand, coil and helix secondary structure types. Bottom panel: The cysteine distribution treated as two separate bivariate distributions based on the oxidation state, with corresponding correlation values. For oxidized cysteines, the correlation values are 0.22, 0.35 and 0.45 for beta strand, coil, and helix secondary structure types. For reduced cysteines, the correlation values are − 0.31, − 0.21, − 0.24

The amino acid cysteine has historically caused substantial inaccuracy in the prediction of amino acid types. Supplemental Fig. 1 shows the wide spread of C_α_ and C_β_ chemical shifts for the cysteine residue distributions over almost the whole expected C chemical shift range for the common amino acids. In contrast, alanine exhibits a tight, well-behaved, unimodal bivariate distribution. The problem of modeling the cysteine distribution as a whole is illustrated by a large bivariate ellipsoid model in Fig. 2b. The broad cysteine residue distribution hinders the use of expected chemical shift values and variances in calculating the probabilities of amino acid types (Wang et al. 2005). The wide cysteine distribution occurs because of the existence of two common side-chain oxidation states for cysteine residues within proteins: the oxidized disulfide-bonded cysteine form and the reduced cysteine form (Sharma and Rajarathnam 2000; Fritzsching et al. 2016). However, while the univariate distributions of individual carbon chemical shifts are broad and indistinct, as shown in Supplemental Fig. 1, the cysteine bivariate chemical shift distributions exhibit distinct modes that are specific to different oxidation states and secondary structure types, as illustrated by multiple contoured density centers in the top graphs of Fig. 3. In contrast, alanine mainly exhibits a single contoured density center for each secondary structure type, as shown in the bottom graphs of Fig. 3. As the calculated C_α_ and C_β_ chemical shift covariances span these extra modes, ignoring them will reduce the amino acid prediction power of the statistical methods utilized in BaMORC. Since the RefDB entries do not indicate the oxidation state of the cysteine residues, we used a K-means clustering method, as described in the Methods, to separate the cysteine residues into two oxidation groups for each secondary structure type, as shown in Fig. 3. We also employed the convention that the ${C}_{o}$ refers to the oxidized form of cysteine while the ${C}_{r}$ refers to the reduced form.

### Refining alpha and beta carbon covariances

**Fig. 4:**
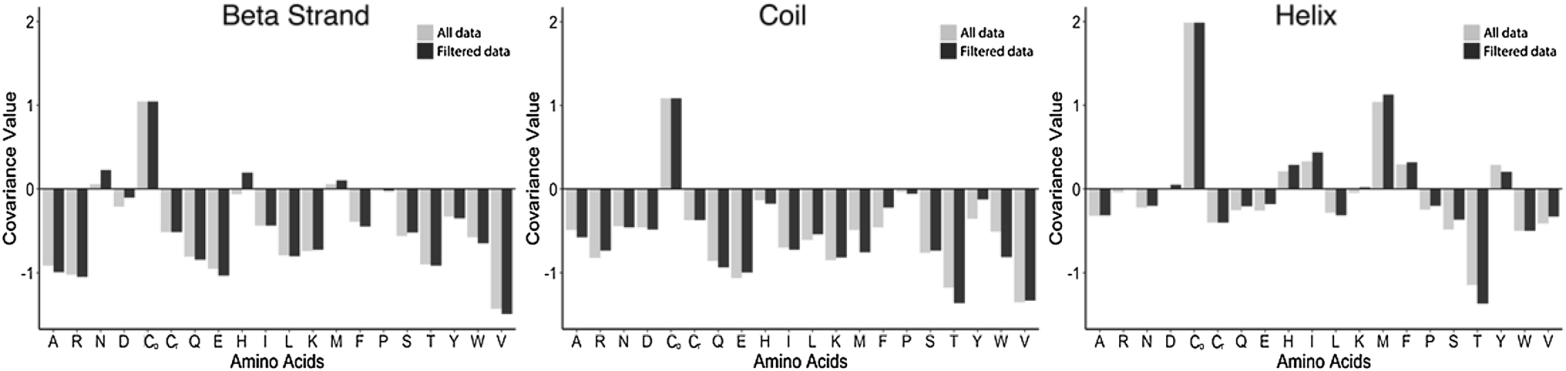
Comparison of covariance values calculated using all of the data from RefDB or using filtered data only. For all three secondary structures, most of the covariances improve, and some of them even show a sign change, which provides a significant improvement of prediction outcomes. Note: the ${C}_{o}$ stands for the oxidized cysteine state and ${C}_{r}$ for the reduced cysteine state

The re-referenced C_α_ and C_β_ chemical shifts in the RefDB are derived from BMRB entries that are based on protein resonance assignments derived from multiple NMR spectra. Unfortunately, it is unclear from a BMRB entry whether a given set of alpha and beta ^13^C chemical shifts are derived from the same NMR spectrum or from multiple spectra, except when assigned peak lists are included, which is the case for only a small fraction of BMRB entries. The C_α_ and C_β_ chemical shifts from different spectra can be misregistered (i.e. shifted out of register with each other), weakening the covariance calculated between these chemical shifts. Therefore, we used quality control measures provided by the RefDB to evaluate the performance of the referencing and to select a subset of entries for deriving amino acid- and secondary structure-specific covariances between C_α_ and C_β_ chemical shifts (see “Methods” for full details). Specifically, we employed the absolute difference between alpha and beta carbon root mean squared deviations (RMSD) from SHIFTX-predicted and observed chemical shifts to order entries. Next, we incorporated entries in a best-first manner into the calculation of C_α_ and C_β_ chemical shift correlations until the sum of the absolute value of these correlations were maximized. After maximization, 729 of the 1557 entries from the RefDB were selected to calculate covariances. The entire workflow is detailed in the “Methods”. In addition, Fig. 4 shows the differences between the covariances calculated before and after optimization. Several of the covariances changed, and these refinements significantly improved the accuracy of the BaMORC methodology as illustrated by the E-Revised matrix results in Fig. 6, Supplemental Fig. 2, and Supplemental Table 3.

### Initial evaluation of different covariance statistical models for unassigned NMR reference correction

We created an unordered pair of C_α_ and C_β_ chemical shifts for a given residue, which we will refer to as a carbon spin system in this context. Unordered pairs were used to test the situation where the amino acid assignment of chemical shifts is not known. Five types of covariance matrices, represented by Matrices A–E, were tested under a generalized chi-squared method to calculate the chemical shift probabilities for each carbon spin system within the BaMORC methodology (see “Methods”). The calculation of variances (sd^2^) and covariances (Cov) are described in Eqs. 6–8 in the “Methods” section. Matrix E utilizes the full set of amino acid- and secondary structure-specific covariances. As mentioned previously, we discovered that the majority of the RefDB datasets are from multiple NMR experiments and are not appropriate for extracting covariance statistics. As described in the Methods, we used the RMSD values of each dataset as a criterion to further filter out datasets that are likely not derived from a single NMR experiment and to develop the Matrix E-revised method. The violin plots in Fig. 6, similar to box plots, but with a visual representation of the full distribution (i.e. a sideways, mirrored histogram), illustrate that the initial Matrix E, which incorporates three separate secondary structure covariances, does not perform well as compared to Matrix D (including the averaged covariance of three secondary structure) and to Matrix B (including no covariance information). The performance is measured on the y-axis (Corrected Reference Value) as a comparison to 568 RefDB datasets treated as a gold standard. This poor performance is due to the use of inaccurate covariances arising from the inclusion of entries that lack the correct correlation between C_α_ and C_β_ chemical shifts, since these shifts may come from separate spectral sources. Matrix E-Revised showed the best performance among the pure statistical models, exhibiting the closest ^13^C reference correction of 0.00 ppm for BMR6032 entry, as shown in Supplemental Fig. 2 and Supplemental Table 3. The performance of Matrix E-Revised as illustrated in Fig. 6 demonstrates the significant improvement in predictions that even small changes in covariances can provide. In addition, for the BMR6032 entry in Supplemental Fig. 2, both the shape of the penalty function to be minimized and the overall minimum value are affected by the type of covariance matrix.

1 $${\Sigma _A}=\left[ {\begin{array}{*{20}{c}} {sd_{\alpha }^{2}}&0 \\ 0&{sd_{\beta }^{2}} \end{array}} \right]$$

2 $${\Sigma _B}=\left[ {\begin{array}{*{20}{c}} {sd_{{\alpha ,i}}^{2}}&0 \\ 0&{sd_{{\beta ,i}}^{2}} \end{array}} \right]$$

3 $${\Sigma _C}=\left[ {\begin{array}{*{20}{c}} {sd_{\alpha }^{2}}&{Co{v_{\alpha ,\beta }}} \\ {Co{v_{\alpha ,\beta }}}&{sd_{\beta }^{2}} \end{array}} \right]$$

4 $${\Sigma _D}=\left[ {\begin{array}{*{20}{c}} {sd_{\alpha }^{2}}&{\frac{{\mathop \sum \nolimits^{} Co{v_{\alpha ,\beta ,~i}}}}{3}} \\ {\frac{{\mathop \sum \nolimits^{} Co{v_{\alpha ,\beta ,~i}}}}{3}}&{sd_{\beta }^{2}} \end{array}} \right]$$

5 $${\Sigma _E}=\left[ {\begin{array}{*{20}{c}} {sd_{{\alpha ,i}}^{2}}&{Co{v_{\alpha ,\beta ,i}}} \\ {Co{v_{\alpha ,\beta ,i}}}&{sd_{{\beta ,i}}^{2}} \end{array}} \right]$$

where *i* is the helix,beta strand,coil.

### Correcting for overlap in amino acid type predictions between statistical models

**Fig. 5:**
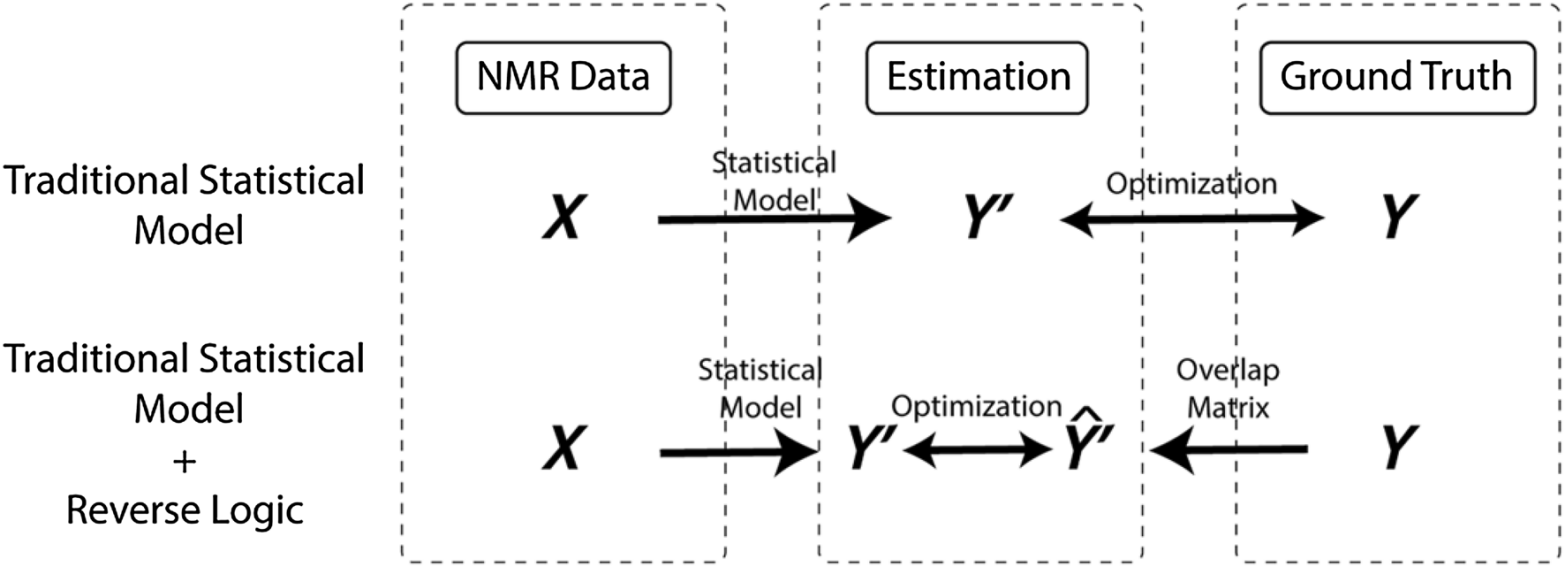
The BaMORC approach with a Bayesian prediction overlap prior matrix. Compared with traditional statistical modeling (top), BaMORC utilizes a reverse logic to employ a prediction overlap matrix as a Bayesian prior to capture the overlap characteristics of the statistical models with respect to the real data. We used data $X$, which are the C_α_ and C_β_ chemical shift values, to predict the normalized amino acid and secondary structure probabilities, which is $Y\text{'}$, via a statistical model. We then multiplied $Y$ by the probability overlap matrix to obtain $\widehat{Y\text{'}}$, mimicking the effects of overlap in the statistical models that are present in $Y\text{'}$

Figure 2 illustrates the substantial overlap of bivariate distributions for a majority of the amino acids. Most statistical learning (SL) algorithms will be biased in favor of certain amino acid types with broad distributions, leading to inaccurate prediction of amino acid and secondary structure types. The standard SL approach estimates an amino acid content frequency$$({Y}^{\text{'}}$$) that is close to the observed amino acid content frequencies ($$Y)$$ via minimizing the difference between $${Y}^{\text{'}}$$and $$Y$$ through specific optimization or search procedures. However, due to the linear relationship limitation, the estimated result $${Y}^{\text{'}}$$ can never eliminate the effects of overlap observed in the amino acid- and secondary structure-specific bivariate distributions in the NMR data. Therefore, we applied a Bayesian-inspired reverse logic to estimate the overlap effects of the C_α_/C_β_ bivariate statistical models on the observed amino acid content frequencies $$Y$$ in order to produce $$\widehat{Y\text{'}}$$. This is accomplished by generating a prediction overlap matrix from the estimated frequency of overlap across C_α_/C_β_ bivariate statistical models using observed C_α_/C_β_ chemical shifts in the RefDB associated with specific amino acid and secondary structure types. The observed amino acid content frequencies $$Y$$ is multiplied by the resulting prediction overlap matrix to produce $$\widehat{Y\text{'}}$$, which mimics the effects of overlap. As an analogy, paper turns yellow from the effects of aging. This aging effect can be mimicked by staining a new piece of paper with tea or coffee and then heating the paper to turn it yellow and make it appear to be old. Likewise, the prediction overlap matrix is mimicking the effects of overlap caused by the statistical modeling. In other words, the prediction overlap matrix acts like a Bayesian prior in estimating the effect of overlap on the observed amino acid content frequencies $$Y$$. This Bayesian-inspired approach is illustrated in Fig. 5 and detailed in the Methods session. Supplemental Fig. 3 shows the prediction overlap matrices for all 20 amino acids. We also employed the diagonal elements of the prediction overlap matrix as weights in the comparison and minimization of differences between $$Y\text{'}$$ and $$\widehat{ Y\text{'}}$$. Thus, the comparison of $$Y\text{'}$$ and $$\widehat{Y\text{'}}$$ utilizes the most discriminating predictors based on prediction accuracy and on the observed prevalence of C_α_ and C_β_ chemical shifts in real datasets. The calculation of the prediction overlap matrix and predictor weights is described in the “Methods”.

**Fig. 6:**
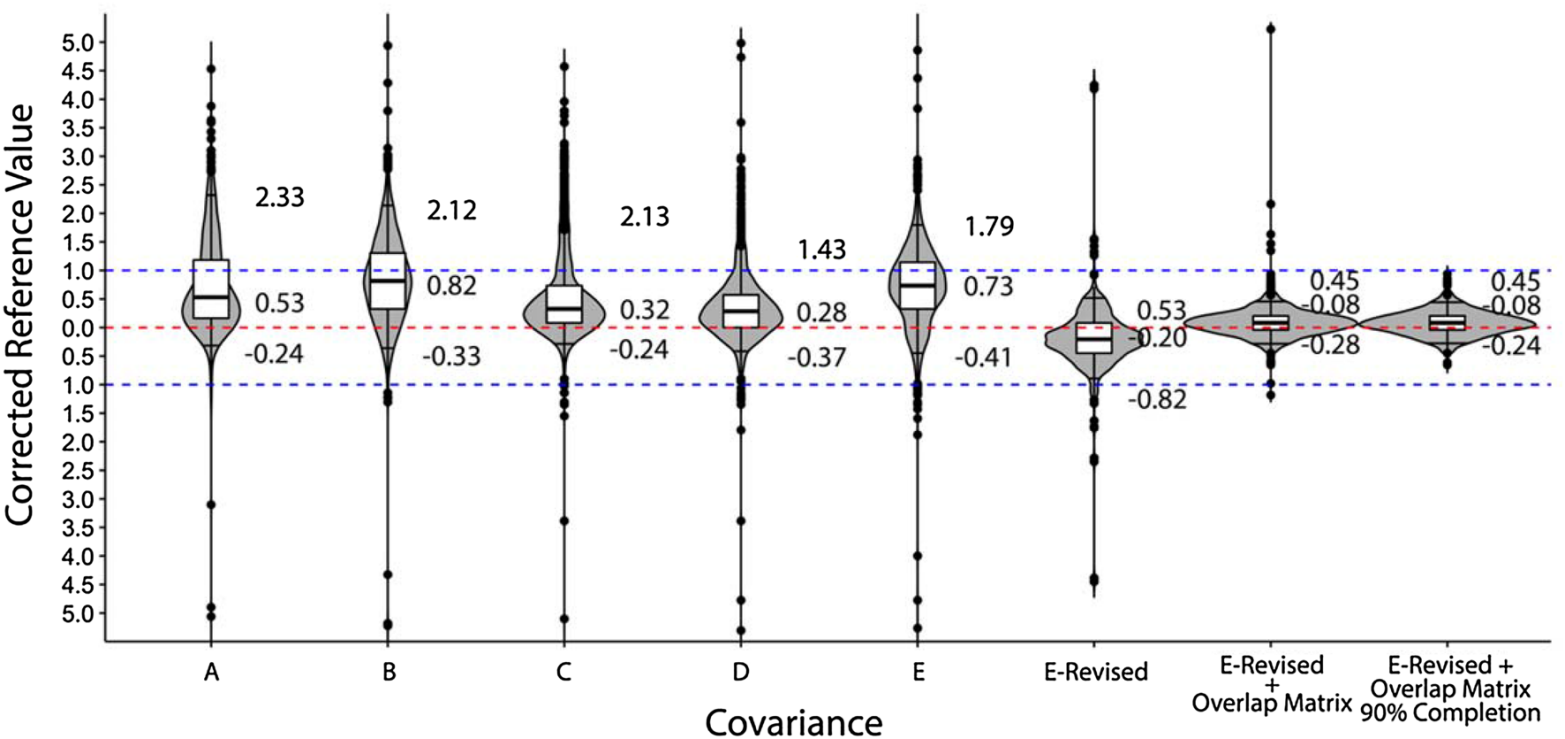
Results across different methods using all RefDB data. Across all of the RefDB data, E-Revised covariance matrix calculated from filtered data performed better. The violin plots here show the distribution of the results. The mark on the top of each plot is the 95% quantile and the one on the bottom is the 5% quantile. The boxplots show the 75%, 50% and 25% quantiles respectively. With both the E-Revised covariance matrix and the Bayesian prediction overlap matrix prior, the algorithm performs the best. Covariance matrices A and C perform similarly, with 90% interquartile ranges (IQRs) of 2.37 and 1.80. Covariance matrices B, D and E show worst results, since their means deviate greatly from the true reference value. The E-Revised matrix performs better, with a 90% IQR of 1.35 and a mean of − 0.20, which is very close to the true reference. After applying the Bayesian prior prediction overlap matrix, the performance of BaMORC shows a dramatic improvement, with a 90% IQR of 0.73 and mean of − 0.08, which far out-performs the state of the art algorithms. When applying the same algorithms on the data with at least 90% completion, the performance of BaMORC remains stable with small improvement, with a 90% IQR of 0.69 and same mean of − 0.08

The BaMORC method combines the E-Revised covariance method used in the chi-squared-based C_α_/C_β_ bivariate statistical models with the prediction overlap matrix, while ignoring glycine residues. The BaMORC method improves the comparison of the predicted and observed amino acid and secondary structure frequencies more than 2.5-fold by modifying Y with the prediction overlap matrix to create $\widehat{Y\text{'}}$, which reflects the overlap introduced by Matrix E-Revised. All of the other statistical models were also tested but performed significantly worse than the BaMORC method, as illustrated by the violin plots in Fig. 6 and Supplemental Table 4. In Fig. 6, we compared the results of reference correction from the set of statistical models based on each covariance matrix (A–E, E-Revised) and the E-Revised covariance matrix with the prediction overlap matrix as applied to all the unassigned RefDB datasets. In this comparison, the E-Revised covariance matrix combined with the prediction overlap matrix acting as a Bayesian prior demonstrated overwhelming performance. The 90% confidence interval was ± 0.45 ppm with an absolute length of 0.73 ppm. When we applied the same approach to the data with at least 90% completion, the BaMORC reference results remain stable with small improvement.

We also tried to add glycine-specific predictors in the BaMORC method. However, the inclusion glycine statistical models had mediocre performance in comparison to using only the 57 non-glycine predictors. This is illustrated in Supplemental Fig. 4, which shows a bimodal distribution of reference correction values with a 90% confidence interval of ± 0.82 ppm and absolute length of 1.64 ppm. The cause of the poor performance appears rooted in the complete overlap of C_α_ chemical shift distributions for beta sheet and coil secondary structure types for glycine residues. This is illustrated by the universally-high prediction-overlap values for glycine predictors as shown in Supplemental Fig. 3. The high values would significantly inflate the product of the matrix multiplication, which will greatly influence the residuals over the range of overlapping C_α_ chemical shift distributions. Thus, in the final implementation of the BaMORC methodology we ignored glycine residues.

### Testing the robustness of the refined NMR shift reference correction method

**Fig. 7:**
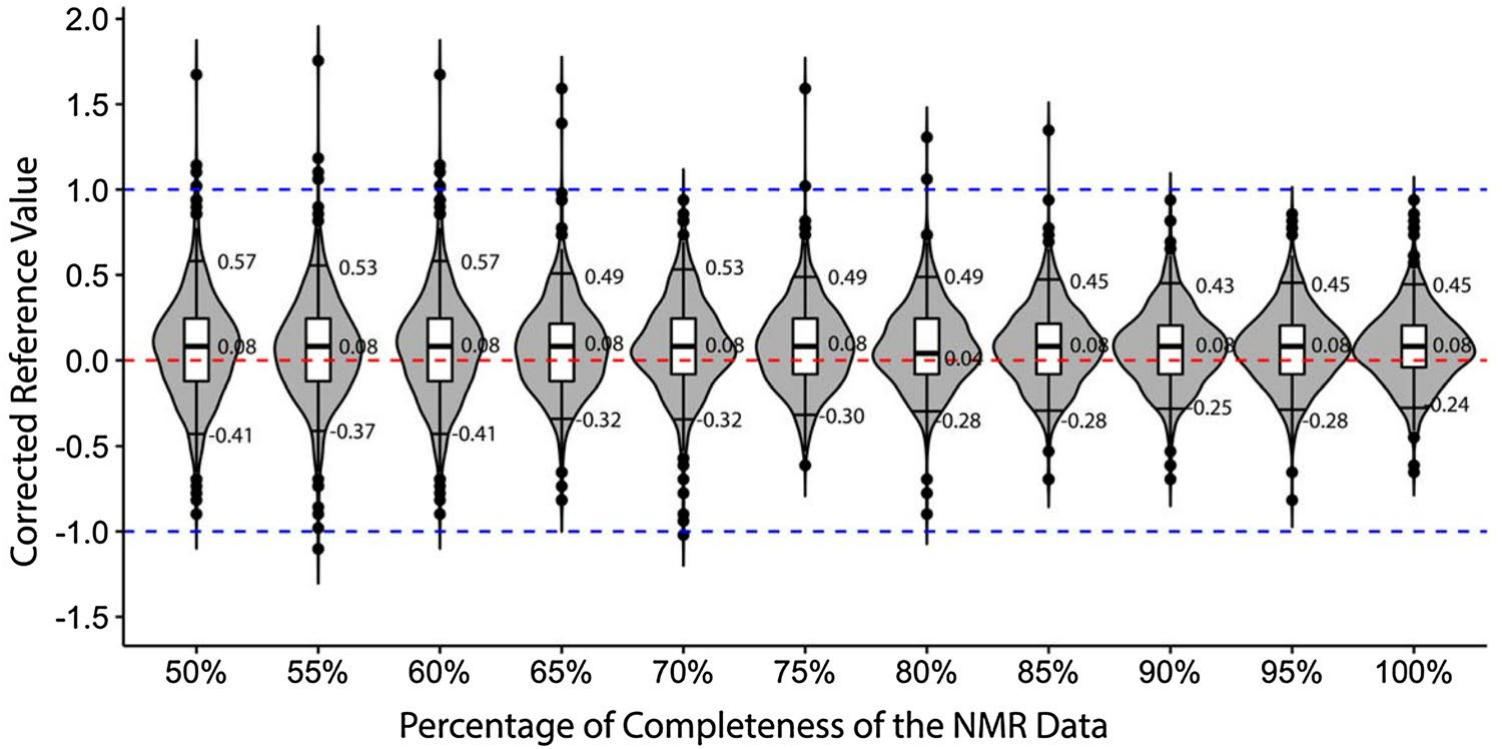
Testing the robustness of BaMORC against varying amounts of missing C_α_ and C_β_ chemical shifts. As dataset completion decreases (from right to left), BaMORC performance decreases only gradually. The violin plots here show the distribution of the results. The mark on the top of each plot is the 95% quantile and the one on the bottom is the 5% quantile. The boxplots show the 75%, 50% and 25% quantiles respectively

Protein NMR datasets are typically incomplete from the perspective of what resonances are expected based on the protein sequence. This incompleteness is due to a host of experimental issues that prevent the detection of all protein resonances. In the RefDB itself, only 568 out of the 1557 entries include 90% or more of the expected C_α_ and C_β_ chemical shifts. Therefore, missing chemical shift data is a real issue that must be addressed. Accordingly, we tested the performance of the BaMORC method using unassigned datasets generated from the RefDB with varying amounts of missing ^13^C spin systems. First, we constructed datasets with 100% completion by removing amino acid sequences for missing C_α_ and C_β_ chemical shift values for 568 entries with 95% or greater starting completion. Then, we incrementally removed 5% of the ^13^C spin systems and tested the performance. Figure 7 and Supplemental Table 5 show the performance when 100–50% of ^13^C spin system data are present. The overall performance of BaMORC does not appreciably deteriorate until approximately 70% of the ^13^C spin systems were missing. Even, with 50% of the spin systems missing, the absolute length of the 90% confidence interval is less than 1 ppm, with the reference corrections within ± 0.6 ppm. Therefore, BaMORC is very robust to missing ^13^C chemical shift data.

### Testing BaMORC with predicted secondary structure

To test the performance of our method in a real-life situation, we removed all of the secondary structure information from the RefDB data and used the sequence-based secondary structure predictions generated from JPred4 (Drozdetskiy et al. 2015). JPred4 is one of the best algorithms for predicting secondary structure from sequence information alone, as showing in Supplemental Fig. 10. We have tried other algorithm also, but JPred Algorithm gives us the best performance: 1258 out of 1557 datasets have a correct prediction percentage of over 70%. Across the RefDB, this breaks down to 46,718 correct helix predictions out of 56,015, 34,063 correct coil predictions out of 73,048, and 34,063 correct beta strand predictions out of 50,930. The new modified version of BaMORC performs as well with the JPred4 prediction as with the “true” secondary structure information from the RefDB, as summarized in Supplemental Fig. 5 and Supplemental Table 6. This result may not be as surprising, since both the SHIFTX and JPred4 methods were developed from structure-based analyses.

### Testing assigned BaMORC versus LACS

**Fig. 8:**
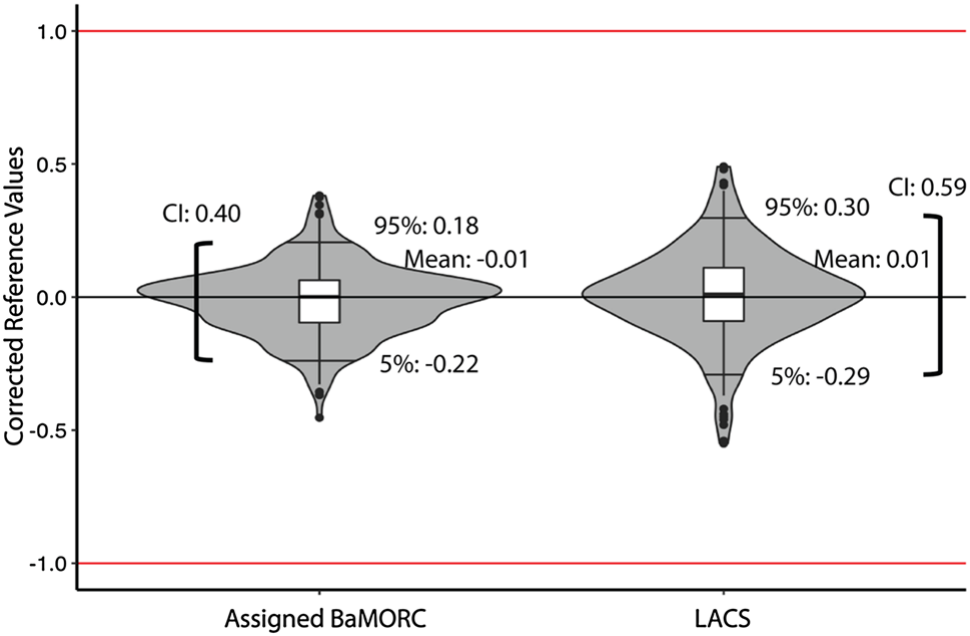
Comparison of Assigned BaMORC versus LACS performance on RefDB. Using known assignment, the Assigned BaMORC with DEoptim algorithm achieve much better results than the LACS algorithm. The violin plots here show the distribution of the results. The mark on the top of each plot is the 95% quantile and the one on the bottom is the 5% quantile. The boxplots show the 75%, 50% and 25% quantiles respectively. The results of Assigned BaMORC (left), it achieved a 0.40 ppm range in confidence interval for data with 90% completion, while LACS achieve slight worse results 0.59 ppm range

While the BaMORC algorithm does not utilize assignment nor structure, we augmented and simplified the base algorithm to utilize assignment information in order to improve reference correction. This alternative implementation called Assigned BaMORC solves the same reference correction problem that the LACS method addresses. Assigned BaMORC takes an assigned NMR-STAR formatted file and returns a single reference offset/correction value for both alpha and beta carbons. We applied Assigned BaMORC and LACS to 1330 datasets from the RefDB with at least 90% assignment completion. On these datasets, assigned BaMORC outperformed LACS as shown in Fig. 8. The 90% confidence interval ranges are 0.41 and 0.59 for Assigned BaMORC and LACS respectively.

### Testing unassigned BaMORC with experimental peak lists

In the case of real-world use, the data obtained from an NMR instrument are not labeled by resonance or grouped into spin systems. To further contribute to the protein NMR field, we applied a new intra-peak-list grouping algorithm developed in our laboratory (Smelter et al. 2017) on top of the BaMORC method and developed a combined method, which we refer to as Unassigned BaMORC. This method can use unassigned three-dimensional HN(CO)CACB-type peak lists to correct the ^13^C chemical shift referencing. This new tool greatly facilitates the automatic analysis and correction of NMR data before downstream analyses. Unassigned BaMORC generates a correction value, a file of re-referenced chemical shifts, and a residual plot showing the optimization of the predicted amino acid frequencies and where the best reference correction value occurs within the optimization. Table 1 shows the performance of Unassigned BaMORC on ten real peak lists derived from solution NMR HN(CO)CACB spectra with secondary structure prediction provided by JPred. These peak lists were manually peak-picked. All ten experimental peak lists have Unassigned BaMORC-predicted reference correction values within ± 0.40 ppm of the RefDB registration offset value, which is better performance than BaMORC’s application across unassigned datasets derived from the RefDB. Two experimental peak lists from BPTI and Z domain of staphylococcal protein A have deviations greater than 2 ppm from the correct carbon chemical shift referencing. Also, none of these experimental peak lists are complete, with several peak lists having over 15% fewer spin systems than expected based on the protein sequence.

**Table 1:** Unassigned BaMORC’s performance on peak lists derived from solution NMR HN(CO)CACB spectra with secondary structure prediction provided by JPred

Protein	Sequence length	Number of spin systems	BMRB ID PDB ID	RefDB Registration offset value	BaMORC Reference correction value	Absolute difference between BaMORC and RefDB
Bovine pancreatic trypsin inhibitor (BPTI) (Vila et al. 2008)	58	47	5359/5PTI	− 8.15	− 8.55	0.40
Cold shock protein (CspA) (Feng et al. 1998)	70	57	4296/3MEF	− 0.06	0.00	0.06
Protein yggU from *E. coli* (Target ER14) (Aramini et al. 2003a)	108	93	5596/1N91	− 0.11	− 0.20	0.09
Fibroblast growth factor (FGF) (Moy et al. 1995)	154	128	4091/1BLD	0.21	0.45	0.24
30S ribosomal protein S28E from Pyrococcus horikoshii (Target JR19) (Aramini et al. 2003b)	82	71	5691/1NY4	0.10	0.25	0.15
Non-structural protein 1 (NS1) (Chien et al. 1997)	73	66	4317/1NS1	0.03	0.41	0.38
Ribonuclease pancreatic (RnaseC6572S) (Shimotakahara et al. 1997)	124	116	4032/1SRN	0.42	0.20	0.22
Ribonuclease pancreatic (RnaseWT) (Shimotakahara et al. 1997)	124	116	4031/1SRN	− 0.18	− 0.25	0.07
Z domain of staphylococcal protein A (Zheng et al. 2004)	71	67	5656/1H0T	2.75	2.69	0.06
Staphylococcus aureus protein SAV1430 (Target ZR18) (Mercier et al. 2006)	91	85	5844/1PQX	− 0.14	0.00	0.14

## Discussion

### Rationale for using RefDB and its limitations

In this statistical model building and data analysis methods development, we utilized RefDB data for several pragmatic reasons. First, the RefDB is the best-referenced large carbon chemical shift dataset that is currently available. Second, we can treat RefDB as a gold standard for evaluation purposes, because it represents a systematic reference correction subset of the BMRB and was the only large dataset we could reasonably use for evaluation of performance. Third, we chose real datasets over simulated datasets, because of the difficulty in generating simulated datasets that represented the complexity of real datasets adequately enough to evaluate performance (Smelter et al. 2017). Simply stated, there was too high a possibility of overestimating performance with simulated datasets that inadequately reflected the complex deviations in carbon chemical shifts of real datasets.

However, it is well recognized in the field that NMR chemical shift data have inaccuracies, and that the RefDB still include errors. Because of these errors, the statistics that we extracted from the RefDB data might not be representative of protein NMR as a whole. Although, a number of algorithms and methods attempt to correct the reference, most of these approaches rely on the assignment of the sequence at the end of the data analysis stage. Our algorithm was built using derived statistics, with the assumptions that the data utilized has been corrected and verified against 3D protein structures, and it makes no attempt to be robust against systematic referencing issues in the SHIFTX method. When analyzing experimental data, it was previously necessary to apply a recursive approach: define a raw reference value; perform the downstream analysis, refine the reference; and repeat the process. Considering these potential artifacts, the statistics that we employed cannot always be directly equated to the true chemical shift statistics of the amino acids present in assigned proteins. Also, RefDB only utilizes chemical shift datasets from proteins with well-defined structure, which means that the BaMORC algorithm is likewise tuned for such datasets.

### Expectations and limitations of the statistical modeling

The underlying statistical modeling implemented in BaMORC also assumes that the ^13^C chemical shifts approximately follow sets of standard distributions. Therefore, the best results are expected when the ^13^C chemical shifts of each amino acid in any secondary structure follow a bivariate normal distribution with no overlap between distributions. We performed normality tests on each chemical shift distribution, which indicated that each distribution was approximately normal and reasonable to be used for parametric statistical purposes in our analysis; however, there is clear overlap between many of these distributions (Fig. 2 and Supplemental Fig. 1). To ameliorate the distribution overlap status quo, we constructed a prediction overlap matrix and predictor weights using a Bayesian-inspired, reverse-logic approach. In addition, amino acid cysteine chemical shift data were classified into two unique distributions to minimize their overlap with other amino acid statistical models, which is justified by the presence of two oxidative states for cysteine residues in the normal cellular environment.

### Bias correction and parameter optimization

During the development of the BaMORC methodology, we addressed several issues regarding chemical shift data quality in the RefDB entries, which are derived from the BMRB. The reference correction of BMRB protein entries provided by the RefDB was a starting point that enabled the derivation of amino acid and secondary-structure-specific expected values and variances for C_α_ and C_β_ resonances. However, we first had to split cysteines into two separate oxidative groups because of overlap problems created by the wide cysteine distributions. Next, problems in inter-spectral registration decouple the assigned chemical shifts reported in the BMRB entries, which are passed onto the RefDB entries utilized in this work. Therefore, we developed several refinements of the RefDB to derive more accurate covariances, improving the performance of BaMORC. To further refine the covariance values, we filtered out all of the datasets that are likely not to come from a single NMR experiment. In the data filtration pipeline described in the Methods, we compared C_α_ versus C_β_ RMSD values of individual RefDB entries. The aim was to use only the entries that represented C_α_ and C_β_ shifts with strong covariance (e.g. derived from single experiments). Among 1557 entries, the correlation optimization filtered down to 729 entries for calculating optimal covariances. The resulting improvement between the inaccurate covariances and the optimal covariances is illustrated in Fig. 4. Nearly all of the 60 covariances are improved, with some showing significant changes including a change in sign. These improvements, as visually illustrated in Fig. 2e, demonstrate the improved accuracy of the resulting statistical models to represent the underlying NMR chemical shift data. Moreover, additional distinct distributions do appear present in Fig. 2 and are due to the presence of other secondary structures and structural phenomena. For instance, it is well-known that cis/trans isomerization of proline has certain effect on secondary structure and affected chemical shift distributions (Schubert et al. 2002). These unaccounted chemical shift distributions can lower calculated covariance values. However, as more BMRB entries include ^13^C-assigned peak lists, we see an opportunity to further refine covariance statistics. According to our estimates, about 180,000 ^13^C-assigned peaks are required in the BMRB for the next generation of covariance analysis. Currently, the BMRB contains approximately 11,500 ^13^C-assigned peaks.

### Reference correction performance on real data

We have tested the performance of the general BaMORC method in detecting reference correction values under various conditions. The reference correction values were within ± 0.45 ppm of the SHIFTX determined references at the 90% IQR with an absolute length of 0.73 ppm for datasets derived from the RefDB. The typical NMR dataset includes approximately 85% of the expected spin systems. Therefore, we tested our algorithm on incomplete data by incrementally removing a certain percentage of the data from each dataset tested. The robustness of the algorithm is stunning: it performs very well, maintaining referencing correction within [− 0.41, 0.57] ppm range of the correct value at the 90% confidence level, even when 50% of the data are randomly removed. This robustness is achieved because the algorithm uses a non-parametric approach (i.e. a comparison of expected and predicted amino acid frequencies). Additionally, keeping reference correction within ± 0.6 ppm of the correct value is very important for accurate amino acid typing used in protein resonance assignment analysis and for accurate secondary structure analysis from chemical shifts. When carbon chemical shift referencing accuracy is outside the [− 0.43, 0.64] ppm range, the relative error rate in amino acid and secondary structure prediction increases dramatically as illustrated by the increase in residuals in Supplemental Fig. 6.

Also, this performance on spin system datasets derived from the RefDB completely translates to the real-world use-case where real, unassigned, experimental HN(CO)CACB peak lists are utilized. All peak list data were manually peak-picked. There are extra peaks in the data, which could be artifacts or from additional resonances due to multiple local protein conformations. Table 1 illustrates even better performance by Unassigned BaMORC on experimental peak lists, keeping chemical shift referencing within ± 0.4 ppm for all ten peak lists tested. While the sample size is small, i.e. only ten experimental peak lists, the superior Unassigned BaMORC performance may reflect the fact that many RefDB derived spin system datasets come from multiple NMR spectra, weakening C_α_/C_β_ correlation and subsequent reference correction performance by BaMORC. Also, two of the experimental peak lists had a carbon chemical shift reference deviation that was over 2 ppm. Peak lists with large chemical shift referencing errors is the exact situation that Unassigned BaMORC was designed to detect and correct, so that a scientist does not waste time and effort trying to utilize such highly miss-referenced peak lists for downstream analyses, especially protein resonance assignment. The resulting assignments would be error prone and their chemical shifts would propagate error during structure determination. But even more subtle deviations in the 0.6–2.0 ppm range can have a significant impact on assignment and structural error. But Unassigned BaMORC has a demonstrated performance in keeping carbon chemical shift referencing within the ± 0.4 ppm range.

### Computational considerations

The computation time is scaled primarily on the number of probabilistic amino acid predictions performed, which in turn is dependent on the size of the data and the number of reference refinement steps; however, there are user-controllable settings allowing a trade-off between accuracy and computation time. By default, 50 incremental steps are employed in both rounds of reference scanning. The first round of scanning is performed over the range of [− 5, 5] ppm in increments of 0.2 ppm, and the second round of scanning is centered at the results from the first round of scanning over the range of [− 1, 1] ppm in increments of 0.04 ppm, resulting in 100 proposals (i.e., proposed references), which for a 10 kDa protein, typically takes approximately 3 min to process on a standard desktop computer. More precisely, the algorithm has a number of stages, each with a different theoretical computational complexity. The grouping stage consists of two steps: registration and spin system grouping. The computational complexity of the registration stage is optimized to $\text{O}({n}^{3}\cdot \text{log}n)$, where $n$ is the number of peaks within the experimental peak list. The average computational complexity of grouping stage is optimized to $\text{O}(n\cdot \text{log}n)$, where $n$ represents the total number of peaks being grouped. At the reference correction stage, each reference proposal requires several matrix multiplications: for each proposal, Unassigned BaMORC performs 60 matrix multiplications for each spin system based on the 20 amino acid types (without glycine and with two cysteine states) and three secondary structure types. The second stage scales linearly with the number of spin systems; however, the constant portion of the computational complexity is significant.

### Model assumptions for appropriate use

An issue facing any model-based approach to data analysis is the validity of the model assumptions. The most important model assumptions here are that each pair of C_α_ and C_β_ chemical shifts is identical and independent, following a bivariate normal distribution, and the shapes of the distribution are well-represented by ellipses. Although we expect the algorithm to be robust to morphologically similar distributions, such as flat-top clusters or low-aspect-ratio ellipses, the algorithm is certainly not designed for the analysis of very small proteins or peptides. In addition, the presence of paramagnetic compounds, ring current effects, and deuteration shift effects will generate outlier chemical shift values that significantly deviate from the expected values derived from the RefDB dataset.

The default assumptions stipulate that each input dataset is at least 50% complete, meaning that the number of missing spin systems should not represent more than 50% of the expected number of spin systems based on the protein sequence. In practice, we found datasets with greater than 70% completion produced consistent reference correction values. If the user wishes to statistically demonstrate the applicability of our approach to a problem, they can employ the residual (sum of the absolute difference) plot. We have thoroughly tested our defaults assumptions on a wide variety of protein scenarios (e.g. all of the relevant entries in the RefDB) and found the correction results to be largely insensitive to protein classification. However, we recognize that there are extreme examples like disordered proteins for which these choices may not be advised. As with all Bayesian analyses, it should be remembered that the prior parameters should genuinely represent the subjective prior beliefs.

### Pragmatic implementation decisions and future development

Unassigned BaMORC is currently designed to correct ^13^C chemical shift referencing using HN(CO)CACB-type peak lists. The focus on ^13^C chemical shift referencing is pragmatic from three perspectives: (i) C_α_ and C_β_ provide the most information about amino acid type, which is central to the BaMORC methodology; (ii) accurate ^13^C chemical shifts have the greatest impact on protein resonance assignment and other downstream analyses; and (iii) grouping of the HN(CO)CACB peaks into spin systems is more robust than for other NMR experiments. Likewise, Assigned BaMORC is designed to use assigned C_α_ and C_β_ chemical shifts for reference correction after initial chemical shift assignment, but before other downstream analyses. However, we are pursuing further improvements to the methodology and current implementations. We see a host of possible improvements that would extend the methodology to correct ^1^H and ^15^N chemical shift referencing and allow the application of the method to peak lists derived from other types of NMR experiments as well. Though, some of the improvements will require further evaluation and refinement of the chemical shifts from BMRB and RefDB entries and may require waiting until sufficient assigned peak lists are present in these public scientific repositories. For instance, developing an extension to handle intrinsically disordered proteins (IDPs) would likely require more than the 176 IDP BMRB entries available as of May 2018.

## Conclusions

The BaMORC method utilizes unassigned C_α_ and C_β_ chemical shift data to generate accurate ^13^C reference correction within ± 0.45 ppm at the 90% confidence level on RefDB derived test datasets. BaMORC also demonstrates robust performance, keeping the ^13^C reference correction within ± 0.6 ppm at the 90% confidence level even with up to 50% of the ^13^C chemical shift data missing. Keeping the reference correction within 0.6 ppm of the correct value is very important for accurate amino acid typing to be used in protein resonance assignment analysis. The Unassigned BaMORC method utilizes unassigned C_α_ and C_β_ chemical shift data from HN(CO)CACB-type experimental peak lists to generate accurate ^13^C referencing correction within ± 0.4 ppm for all 10 HN(CO)CACB-type experimental peak lists tested. The Assigned BaMORC method utilizes assigned C_α_ and C_β_ chemical shift data to generate accurate ^13^C chemical shift reference correction within ± 0.22 ppm at a 90% confidence interval. Unassigned BaMORC can correct ^13^C chemical shift referencing at the beginning of protein NMR analysis, when accurate ^13^C chemical shift referencing is needed the most for accurate protein resonance assignment, structure determination, and other downstream analyses. Assigned BaMORC can refine the referencing once assignments are made. Additionally, the underlying BaMORC method is robust to missing ^13^C chemical shift data, which addresses the real-world situation of incomplete ^13^C resonance detection. Therefore, the BaMORC methods will allow non-NMR experts to detect and correct ^13^C referencing error at critical early data analysis steps, lowering the bar of NMR expertise required for effective protein NMR analysis.

## Methods

### Datasets preparation

We downloaded the 2162 available protein chemical shift datasets from the Re-referenced Protein Chemical shift Database (RefDB)^1^ on May 4th, 2015 (Zhang et al. 2003). The developers of the RefDB have carefully corrected the referencing of ^1^H, ^13^C, and ^15^N chemical shifts in BioMagResBank (BMRB) entries using the SHIFTX-predicted chemical shifts based on corresponding 3D protein structures in the Protein Data Bank (PDB), which is managed by the international collaboration known as the worldwide Protein Data Bank (wwPDB) (Berman et al. 2006). Among the 2162 RefDB entries, we employed 1557 that contained both C_α_ and C_β_ chemical shifts, both to derive the necessary statistics and then to subsequently test our methods. Secondary structure specific information was likewise downloaded and extracted from the RefDB website.

For each RefDB entry, we first parsed the text data files with the extension of “.str.corr”, which are mostly in NMR-STAR 2 format, with additional sections added by RefDB, with a short R script that uses crafted regular expressions to clean and convert the relevant assigned chemical shift data into a tab-based format for parsing. The reason for this conversion step is to remove unnecessary metadata, missing values, blank spaces, and section breaks. In this conversion, we retained the full sequence, residue position, amino acid typing, secondary structure, and C_α_ and C_β_ chemical shift information. Statistics were also calculated from the resulting data and verified using the results reported in the RefDB. Based on amino acid and secondary structure, we subdivided the data into 60 classes based on 20 amino acid types and three secondary structure types. In the early part of the methods development, we ignored the glycine classes and only employed the other classes representing the 19 amino acids with C_β_ resonances.

### K-means clustering of oxidized and reduced cysteine alpha and beta carbon chemical shifts

From Fig. 3, we concluded that cysteine chemical shifts are too broad and needed to be treated as two different populations based on two oxidation states, reduced and oxidized. For this purpose, we utilized the K-means clustering machine-learning algorithm (Endo and Miyamoto 2015). This algorithm requires the expected number of clusters, K, which was two in this specific application. The algorithm begins by selecting K = 2 data points as “centroids” and groups each C_α_–C_β_ pair into two clusters based on the smallest Euclidean distance from cluster centroids. Then, it uses iterative techniques to re-calculate the centroids and re-group the data until the centroids converge. To verify the clustering results, we compared the means and standard deviations of the two new subgroups with statistics reported in the RefDB. It is worth mentioning that even though the statistics from RefDB included two-state cysteines, there are no labels on any specific cysteine in the RefDB NMR data.

### Calculating and refining alpha and beta carbon covariances

After grouping all of the RefDB datasets based on amino acid and secondary structure, we calculated the covariance between C_α_ and C_β_ for each group. We first calculated the mean ($\mu$) and standard deviation (sd) for C_α_ and C_β_ of each group *i*, as show in Equation sets 6 and 7. Then, we used Eq. 8 to calculate the covariance Cov_α,β_.

6 $${\mu _\alpha }=\frac{{\mathop \sum \nolimits_{{i=1}}^{n} {C_{\alpha ,i}}}}{{n}};~{\mu _\beta }=\frac{{\mathop \sum \nolimits_{{i=1}}^{n} {C_{\beta ,i}}}}{{n}}$$

7 $$s{d_\alpha }=\sqrt {\frac{{\mathop \sum \nolimits_{{i=1}}^{n} ({C_{\alpha ,i}} - {\mu _\alpha })}}{{n - 1}}} ;~s{d_\beta }=\sqrt {\frac{{\mathop \sum \nolimits_{{i=1}}^{n} ({C_{\beta ,i}} - {\mu _\beta })}}{{n - 1}}}$$

8 $${Cov_{\alpha ,\beta }}=~\frac{{\mathop \sum \nolimits_{{i=1}}^{n} \left( {{C_{\alpha ,i}} - {\mu _\alpha }} \right)\left( {{C_{\beta ,i}} - {\mu _\beta }} \right)}}{{n - 1}}$$

The covariance matrix was constructed using Eq. 9 and the matrix representation was employed in the algorithm.

9 $$\Sigma =\left[ {\begin{array}{*{20}{c}} {sd_{\alpha }^{2}}&{Co{v_{\alpha ,\beta }}} \\ {Co{v_{\alpha ,\beta }}}&{sd_{\beta }^{2}} \end{array}} \right]$$

**Fig. 9:**
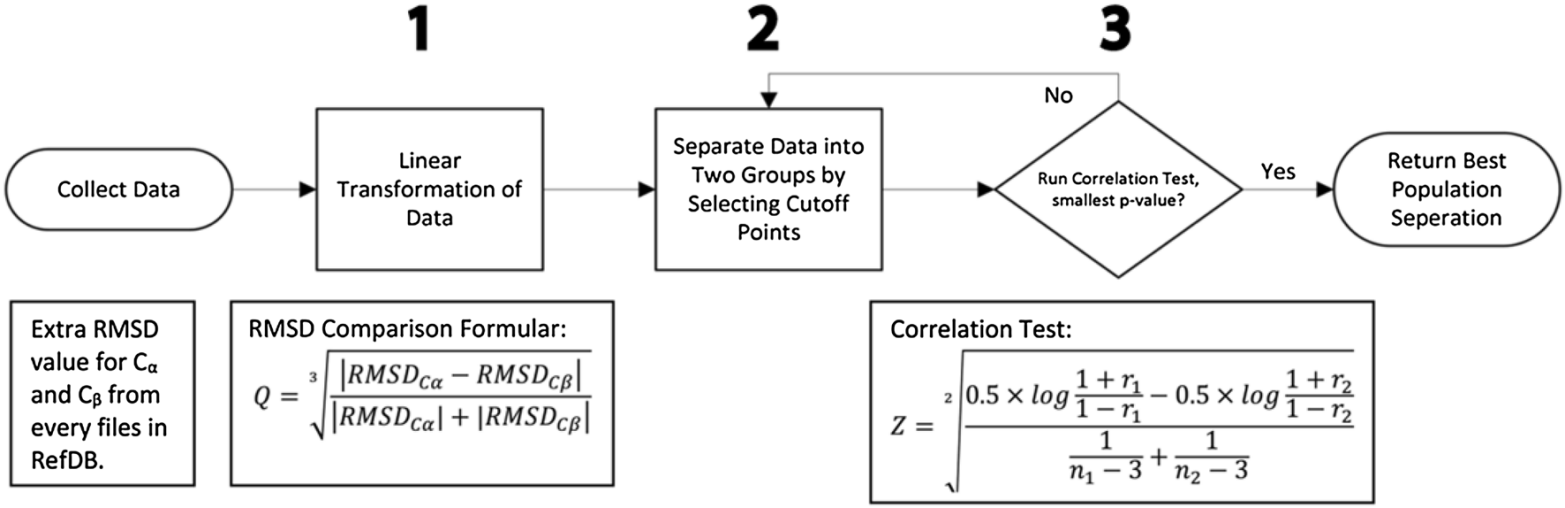
Data selection algorithm for re-calculating covariances. Based on the RMSD values accompanied by RefDB datasets, we first (1) performed a cubic root transformation; then (2) separated the datasets into two groups based on the Q values calculated from the (1) and recalculated covariance for each group; and (3) ran a correlation test to justify whether the usability of one subgroup based on 3 criteria: a small RMSD difference, a small Q value, and a small p-value against the other subgroup. We then repeated steps (2) and (3) to identify the subgroup with the best sample for covariance calculations

Due to the variation in the quality of the data, the covariances calculated from all of the RefDB data are not representative, causing the reference correction values to be less accurate. When C_α_ and C_β_ chemical shift data are collected from two separate NMR experiments, two independent samples of chemical shifts are generated. Similar to the batch effects, these two samples are independent and the correlation between the ${\upalpha }$ and $\beta$ carbons are weakened or even destroyed. Thus, it was necessary to select a subgroup of data and re-calibrate the covariance. The data filtration procedure is shown in Fig. 9.

We employed the root mean squared deviation (RMSD) as the criterion for selecting subgroups. The RMSD is recorded in every data file in the RefDB. The RMSD is a measurement of the confidence interval of the population mean (mean of the difference between the calculated and observed shifts) for each single data point. This statistic is calculated from Student’s t-test. The higher the RMSD value, the less accurate the corrected data. In our methodology, we have two RMSDs from the C_α_ and C_β_ nuclei. To select the best datasets, we need lower individual RMSDs, a smaller difference between the two RMSDs, and, simultaneously, the maximum difference in the correlation between two subgroups (useful data and non-useful data). Thus, we first compared the two RMSD values, using the RMSD comparison equation Q, as shown in Fig. 9. The rationale behind this transformation is the minimization of the difference between RMSDs, which is the absolute difference in the numerator under the cubic root, and the minimization of individual RMSD values by dividing the numerator by the sum of their absolute values. In this context, the cube root is a standard statistical transformation method, allowing a very skewed distribution to approximate a normal distribution (Wilson and Hilferty 1931; Krishnamoorthy et al. 2012), as shown in Supplemental Fig. 9. Then, we divided the data into two groups based on the cutoff point from the Q values, calculated the correlations ${r}_{1}$ and ${r}_{2}$ of both groups, and then used the correlation test to calculate the p-value, as shown in steps 2 and 3 of Fig. 9. By recursively applying steps 2 and 3, we identified the smallest p-value as the final cutoff point. All of the data that provide Q values smaller than the cutoff point is included in the datasets to further refine the covariance.

### Calculating the overlap matrix and classifier weights

Sixteen of the 19 amino acid C_α_–C_β_ bivariate distributions overlap almost completely, as shown in Fig. 2. Due to the linearity of the statistical model, our methodology will favor those amino acid and secondary structure types with broad distributions and lead to over-prediction of those types. To side-step this problem, we applied a Bayesian-inspired reverse logic approach on top of the traditional statistical model. In the traditional model, we used data $X$, the C_α_ and C_β_ chemical shift values, to predict the ${Y}^{\text{'}}$(the normalized amino acid and secondary structure probabilities), which can be simplified as the estimated amino acid types composition. To calculate the reference value, the model minimized the difference (sum of the absolute difference) between ${Y}^{\text{'}}$and $Y$(the normalized amino acid and secondary structure frequencies), which can be simplified as actual amino acid types composition, through grid-searching. We then multiplied the probability overlap matrix by $Y$ to calculate $\widehat{Y\text{'}}$, thereby turning a discrete classification into a “fuzzy” classification and capturing the overlap characteristics of the data. To calculate the $\widehat{ Y\text{'}}$, we used the following equation: $\widehat{ Y\text{'}}=Y\times {\varOmega }_{overlap}$. Since we considered three secondary structure types here, the dimensions of both $\widehat{Y\text{'}}$ and $Y$ were $1\times 57$, and the ${\varOmega }_{overlap}$ is a $57\times 57$ matrix. When considering glycine, a $3\times 3$ overlap matrix was employed. Finally, we concatenated the three glycine results into the 57-element vector to form a new $\widehat{Y\text{'}}$ and $Y$ with $1\times 60$ dimensions. The prediction overlap matrix calculation is based on probability calculations derived from each of the 60 statistical models. On the basis of amino acid types (excluding glycine) and secondary structure, we first grouped all of the chemical shifts into 57 bivariate groups/classes and 3 univariate groups/classes for glycine. Then, for every pair of C_α_ and C_β_ chemical shifts, we calculated the probabilities of the 57 classes. Likewise, we used every glycine C_α_ chemical shift to calculate the probabilities for the three glycine classes. For example, for every data point of an alanine-beta strand, we calculated the probabilities of all of the classes. Then, we performed normalization across the columns and finally obtained a $57\times 57$ matrix.

In nature, amino acid chemical shift distributions are not ideal; i.e., the C_α_/C_β_ bivariate statistical models approximate the real distributions. Hence, we used the real distributions to calculate the prediction overlap between the bivariate statistical models and represented this overlap as prior information in the form of a prediction overlap matrix. Moreover, we employed the diagonal elements of this matrix (Supplemental Fig. 3) as weights$({\omega }_{i}$’s), in the calculation of residuals. This maximizes the use of classifiers with the least overlap and, thus, the best prediction performance.

The overall optimization approach can be simplified into the following residual equation which is minimized as showing in Eq. 10.

10 $$\hbox{min} \left( {\mathop \sum \nolimits^{} {\omega _i}\left| {Y_{i}^{\prime } - \hat {Y}_{i}^{'}} \right|} \right)=\hbox{min} \left( {\omega ~ \cdot \left| {Y^{\prime} - Y \cdot {\Omega _{{\text{overlap}}}}} \right|} \right)=\hbox{min} \left( {\left| {\omega ~ \cdot Y^{\prime} - \omega \cdot Y \cdot {\Omega _{{\text{overlap}}}}} \right|} \right)~$$

To calculate the $\widehat{Y\text{'}}$, we multiplied $Y$, the ground truth, with the overlap matrix, ${\varOmega }_{overlap}$. This $\widehat{Y\text{'}}$ captures the overlap characteristics of the statistical models with respect to the data. Then, to best utilize the statistical models with the best predictive power, we further multiplied $Y\text{'}$ and $\widehat{Y\text{'}}$ by the weights, $\omega$. By utilizing a grid-searching method, we identify an optimal value that minimizes the absolute difference between the outcomes from both the estimated and actual amino acid and secondary structure compositions.

### BaMORC methodology

**Fig. 10:**
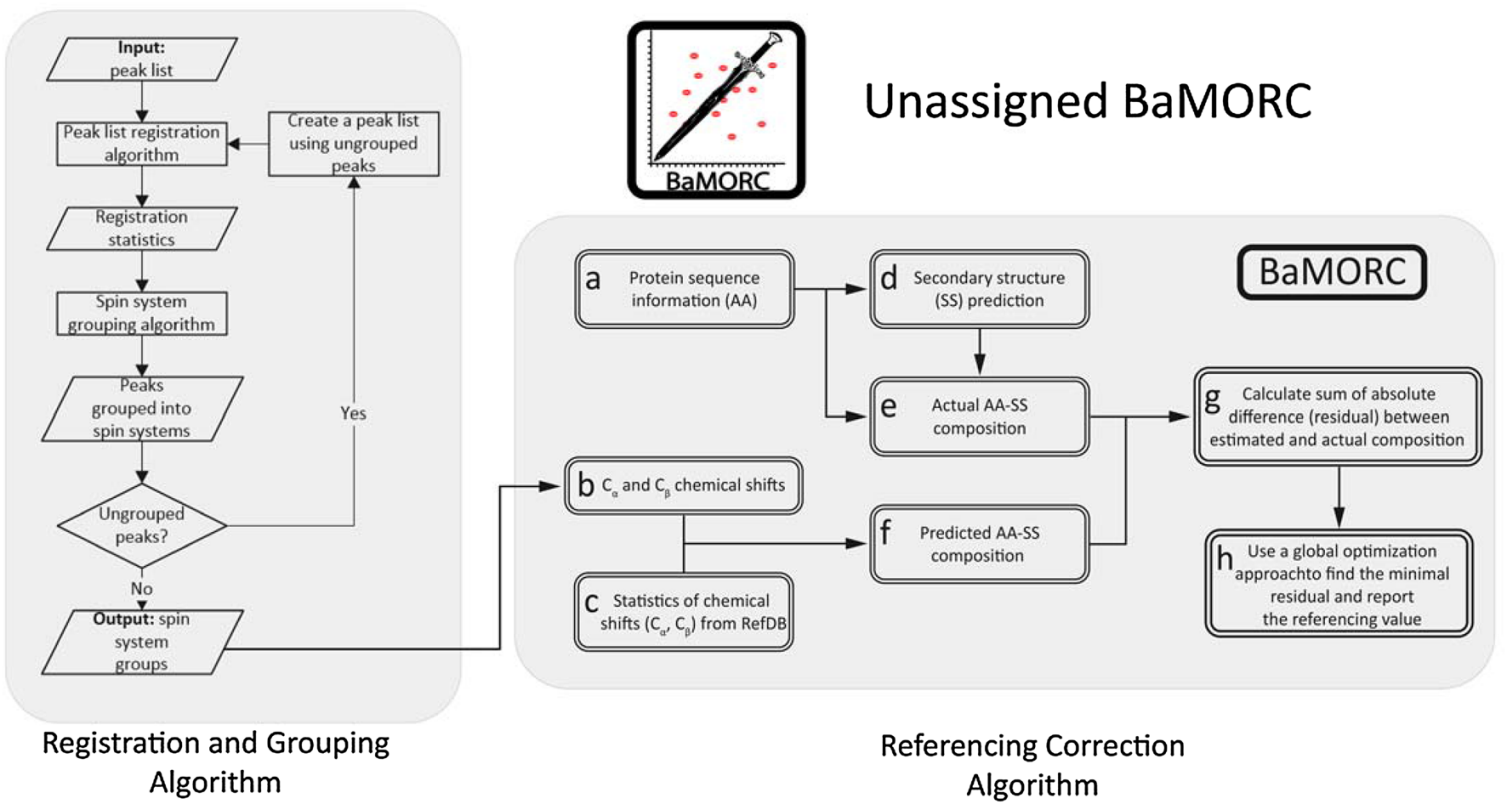
Flow diagram of the Unassigned BaMORC method. Unassigned BaMORC includes two algorithmic parts: grouping and reference correction. The grouping algorithm utilizes the density-based clustering algorithm DBSCAN to group peaks and report C_α_/C_β_ spin systems as the input for the correction algorithm BaMORC, which includes estimate amino acid composition, secondary structure prediction and optimization of the absolute difference between the estimated and actual amino acid composition, to report a reference correction value as the final output

The bottom right flowchart in Fig. 10 provides an overview of the BaMORC method. In describing this method, let ${V}_{AA,SS}$ denote the chemical shifts space. ${V}_{AA,SS}=\left({X}_{{c}_{a},1}, {X}_{{c}_{b},1}\right), \dots , \left({X}_{{c}_{a},2},{X}_{{c}_{b},2}\right)$, where $AA\in \left(19 \ amino \ acid \ types\right)$ and $SS \in \left(3 \ secondary \ structure \ types\right)$. We exclude glycine here for simplicity, since it does not have a beta carbon. The reference correction method assumes that for each ${V}_{AA,SS}$, it follows a unique bivariate normal distribution. For example, ${V}_{Alanine, Helix}~MVN({\mu }_{{c}_{a}, A,H}, {\mu }_{{c}_{b}, A,H}, {\varSigma }_{A,H})$, whereby a covariance ($\varSigma$) exists between and the $\alpha$and $\beta$ ^13^C chemical shifts. To calculate the probability, we first need to transform each pair of the chemical shifts to a Chi square value using Eq. 11, and ${\chi }^{\text{*}}$ follows a Chi square distribution with 2 degrees of freedom ${\chi }_{2}^{2}$ (for glycine, ${\chi }_{1}^{2}$). But in the final version of our method, we removed glycine models based on robustness testing. Then, we can calculate the probability of each of the amino acid type and secondary structures for any pair of $\alpha$ and $\beta$ ^13^C chemical shifts. For a given NMR dataset with $n$ pairs of chemical shifts, the BaMORC will calculate 57 possibilities for each pair of chemical shifts and 3 possibilities for single chemical shifts, among which the maximized probability represents the corresponding amino acid type and secondary structure. The BaMORC method computes every probability across the dataset, sums up them based on amino acid type and secondary structures, and then normalizes the sums so that the sum of the sums is equal to 1. These 57 sums represent the estimated composition frequency. The difference between the estimated composition and the actual composition, which is calculated from the sequence, is minimized via a grid search. The assumption is that the dataset with the correct reference should report the lowest difference, as the two compositions should match closely.

11 $${\chi ^{\text{*}}}=\left[ {v - \left( {{{\hat {\mu }}_{{c_a},AA,SS}}{\text{~}}{{\hat {\mu }}_{{c_\beta },AA,SS}}} \right)} \right] \times \Sigma _{{AA,SS}}^{{ - 1}} \times {\left[ {v - \left( {{{\hat {\mu }}_{{c_a},AA,SS}}{\text{~}}{{\hat {\mu }}_{{c_\beta },AA,SS}}} \right)} \right]^T}$$

The search range is typically limited to − 5 to 5 ppm centered around the current reference value of 0. The algorithm first evenly samples 50 candidate reference correction values in the range from − 5 to + 5. Each of the candidate values is applied in the whole dataset, and the difference between the estimated and actual amino acid composition frequency is calculated. The one value that minimizes the difference is the raw correction value, ${M}_{1}$, and then around this value the algorithm will evenly sample another 50 candidates around this value, from the range between ${M}_{1}-1$ and ${M}_{1}+1$. The algorithm subsequently performs the same calculation to identify the value that minimizes the difference and reports it as the final correction value, ${M}_{2}$. To further reduce the computational time, we also utilized global optimization algorithm(Mullen et al. 2009) to estimate the referencing correction value, which has the similar methodology behind the scene as the grid search approach, and the results is shown in the Supplementary Fig. 7. We tested three max iteration numbers for the global optimization DEoptim function: 10, 20, 50. The results from these three settings are very similar, with the hither iteration value, the results get better trivially but computational time increase exponentially, which is from > 2 min to < 15 min per dataset.

### Assigned BaMORC method

The assigned BaMORC approach uses the assigned amino acid type information along with secondary structure prediction from JPred to greatly reduce the number of amino acid typing probabilities that are calculated, i.e. from 60 probability calculations for each C_α_/C_β_ pair in BaMORC to only 1 probability calculation (step f in Fig. 10). Also, the resulting optimization problem has a smooth enough error surface to use better optimization methods than a grid search (Supplemental Fig. 8). Therefore, we included the global optimization by differential evolution (DEoptim) (Mullen et al. 2009). Both improvements together decrease the running time of the method to less than 1 min. The comparison of BaMORC performances using grid search optimization vs global optimization was shown in Supplemental Fig. 8. In an essence, the global optimization computational timing is shorter with a better performance for NMR data with assignment results.

### Unassigned BaMORC method

Conceptually, the algorithm consists of two parts. A full schematic representation of the analysis workflow is provided in Fig. 10. The first part of the Unassigned BaMORC method groups the peaks in the 3D HN(CO)CACB peak list into spin systems using ^1^H and ^15^N common resonances (Smelter et al. 2017). Ideally, the HN(CO)CACB peak list will contain two peaks for every amino acid except for glycine, which lacks a beta carbon, so the number of spin system groups in the HN(CO)CACB peak list will be equal to the number of amino acids minus the number of glycine residues. The second part of the Unassigned BaMORC method uses the ^13^C_α_ and ^13^C_β_ carbons chemical shifts for every spin system group returned by the grouping algorithm and employs the BaMORC method to calculate and return the carbon reference correction value.

#### Grouping methodology (spin system grouping algorithm)

The spin system grouping algorithm, as illustrated in Fig. 10, can group peaks into spin systems in peak lists that have multiple peaks per spin system. In this use-case, the HN(CO)CACB NMR peak list contains two peaks for each spin system group except for the glycine residues. The grouping of peaks into spin systems is complicated by the presence of multiple sources of variance in dimension-specific peak positions; i.e., different dimension-specific match tolerance values are necessary to reliably group peaks into spin systems without overlap. Our grouping algorithm consists of two parts: the registration step and the actual grouping step (Smelter et al. 2017). The registration step derives the necessary match tolerance values from the single-peak lists necessary to group peaks into spin systems. The grouping algorithm is based on the widely-used density-based clustering algorithm DBSCAN (Ester et al. 1996) and employs derived dimensions-specific match tolerances values to group peaks into spin systems. It uses a Chi square distance cutoff and variance-normalized distance (Chi square value) to decide whether the peaks can be grouped into spin systems. To address the problem of multiple sources of variance, the algorithm is developed in an iterative fashion, which allows it to readjust match tolerance values in the case where peaks are left ungrouped by repeating the registration step again and grouping as many peaks into spin systems as possible. Figure 10 is the flow diagram of the iterative grouping algorithm. First, the grouping algorithm reads in a single peak list in and runs the registration inn order to identify the initial match tolerances for each comparable dimension (for ^1^H and ^15^N in the case of HN(CO)CACB), for ^1^H and ^15^N. Next, it groups peaks into spin system clusters using the derived match tolerance values. Then, the algorithm checks whether any ungrouped peaks remain and, if so, creates a new peak list and attempts to register it again itself again to determine new, larger match tolerances that can be used to group peaks into spin systems.

#### Reference correction methodology (BaMORC)

The reference correction methodology is essentially BaMORC. The input of the algorithm is the output from the grouping methodology, which are pairs of ^13^C chemical shifts derived from pairs of grouped HN(CO)CACB peaks. Using these pairs of ^13^C chemical shifts and the same BaMORC analysis pipeline reports an optimized correction value as a reference. Eventually, Unassigned BaMORC applies this correction value to all of the C_α_ and C_β_ chemical shifts and prints out a text file, that including all of the corrected peak lists in the final output.

## Electronic supplementary material

Below is the link to the electronic supplementary material.

Supplementary material 1 (DOCX 3962 KB)
