# Supplementary material for: Automatic 13C chemical shift reference correction for unassigned protein NMR spectra
Source: J Biomol NMR. 2018 Aug 10;72(1):11–28. doi: 10.1007/s10858-018-0202-5 (PMC6209040; doi:10.1007/s10858-018-0202-5)
Supplement: Supplementary file 1 — Supplementary material 1 (DOCX 3962 KB) [file 10858_2018_202_MOESM1_ESM.docx]

**Supplemental Figures**

**
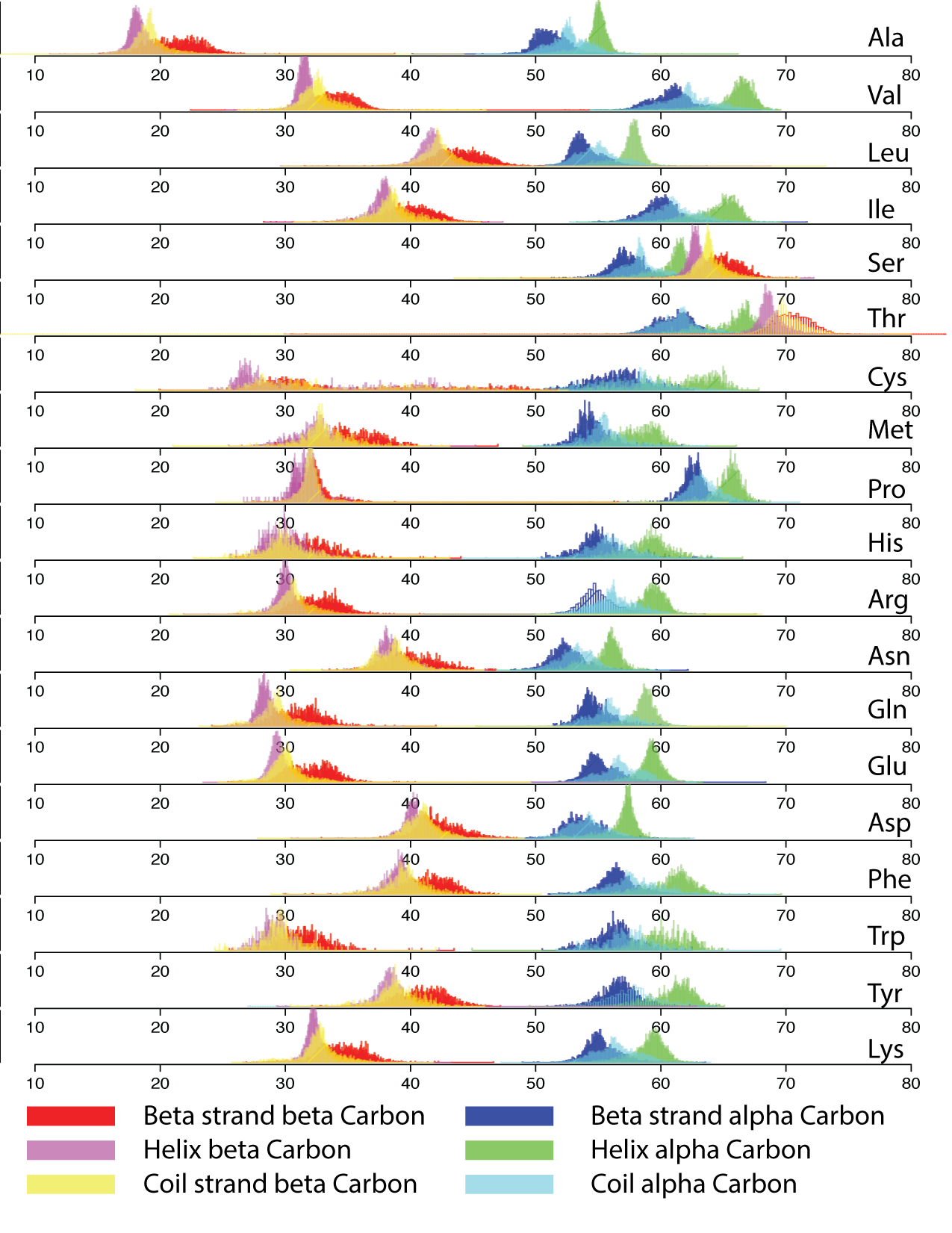
**

**Supplemental Figure 1. 1D Distribution of alpha and beta carbon chemical shifts specific to the amino acid and secondary structure types.** Each of the 19 amino acids exhibits at least 3 pairs of chemical shift distributions, corresponding to beta strand, helix and coil secondary structure types. Most distributions show alpha carbon at high chemical shift values (45-70 ppm) and beta carbon at low chemical shifts (10-45 ppm), except for serine and threonine. Cysteine presents the broadest distribution.

**
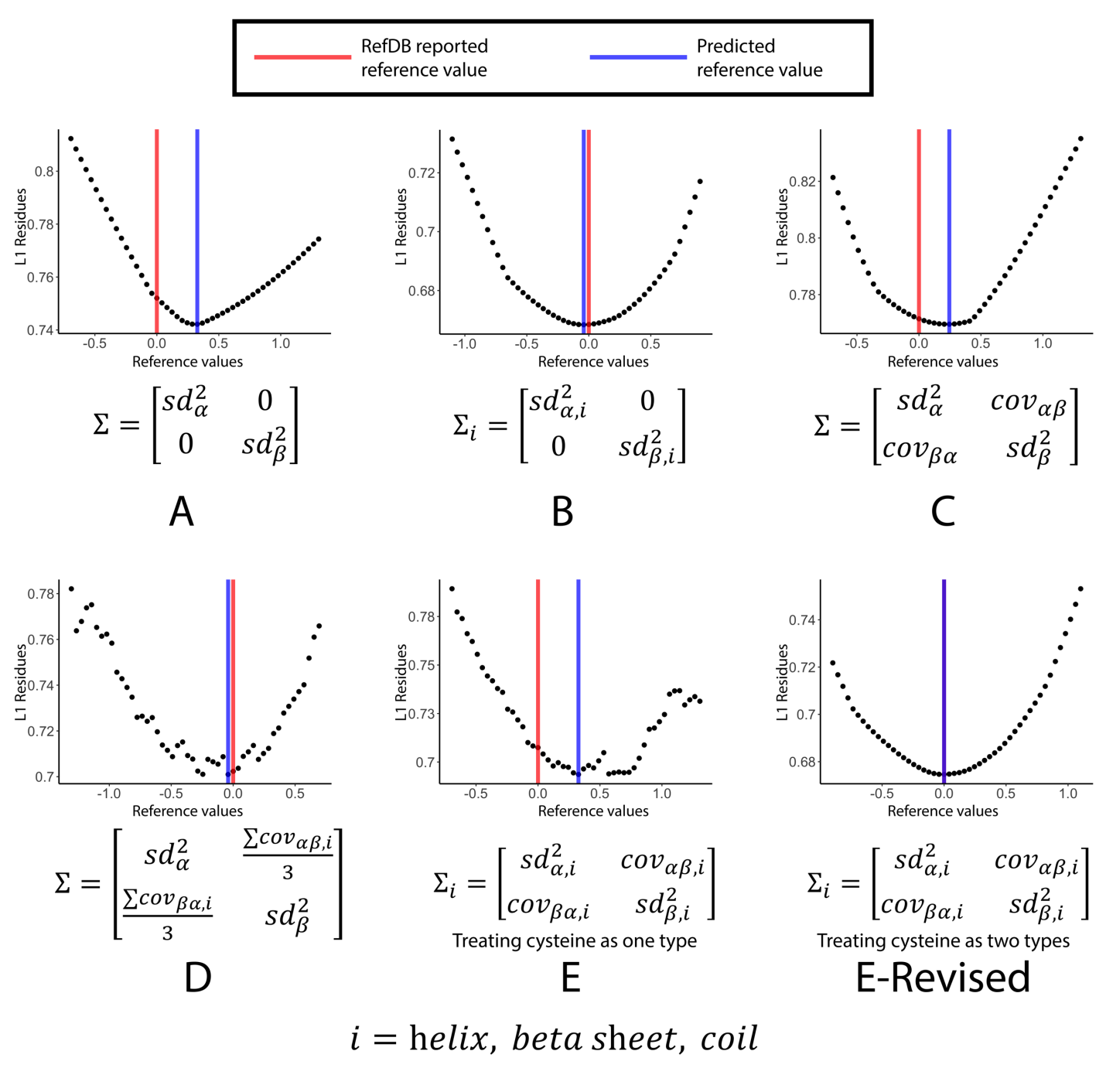
**

**Supplemental Figure 2. Performance of different covariance matrices on the BMR6032 data.** All panels show step-wise plots of the second 50-step grid-search, with the corresponding covariance matrix presented below. The covariance matrices A, C and E all show a major deviance from the true reference value. Matrices B and D perform equally well but with small deviance. The E-Revised matrix performs the best, with its output exactly matching the true reference value.

**
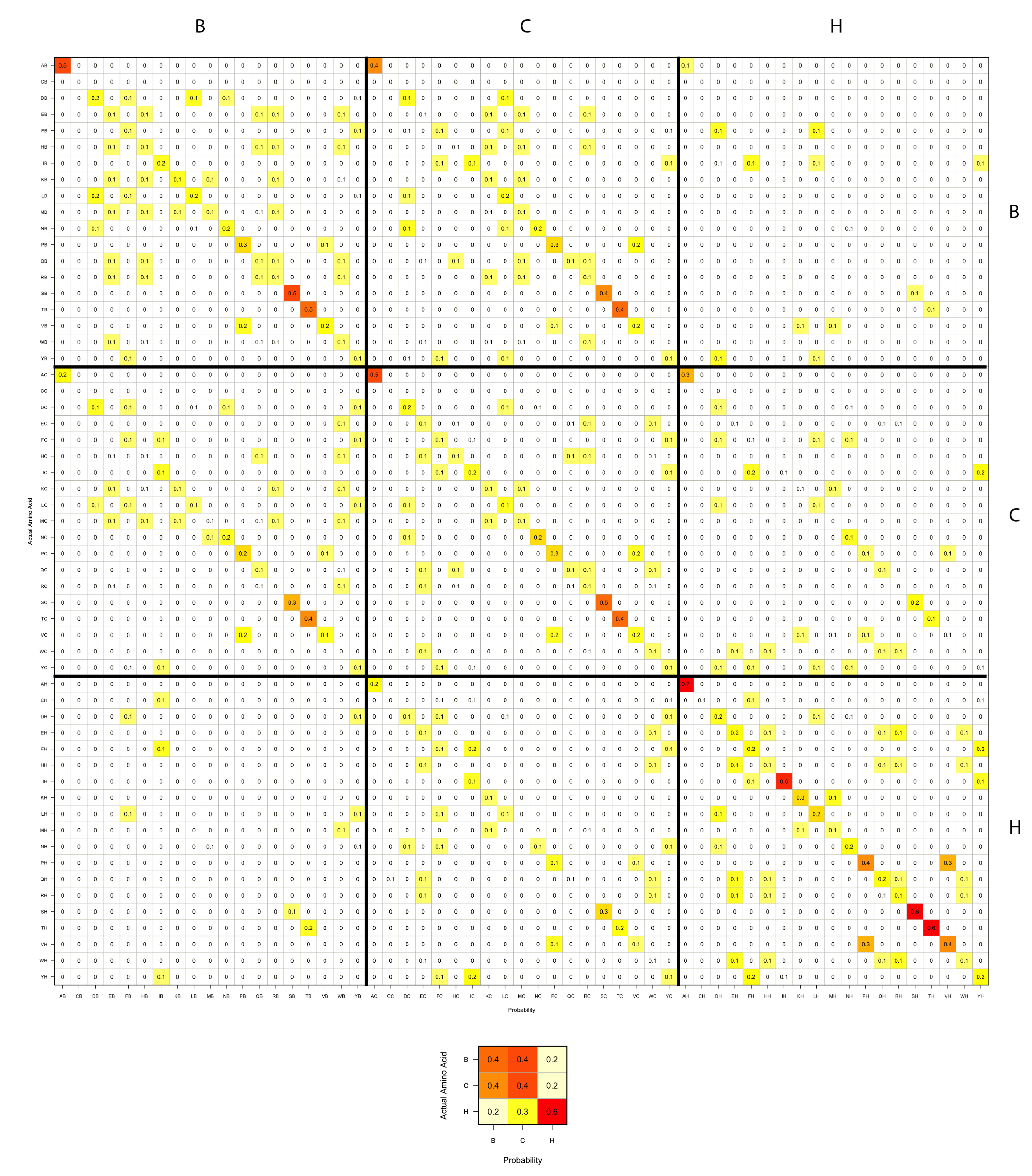
**

**Supplemental Figure 3. Bayesian prediction overlap prior matrix derived from the bivariate statistical models and chemical shifts from the RefDB.** Top: Probability overlapping matrices for amino acids excluding glycine. Bottom: Overlapping matrix for glycine. The color represents the value in the matrix: a higher value corresponds to a darker red color, and a lower value to a light yellow. Higher diagonal probabilities indicate better predictive power of the given model.

**
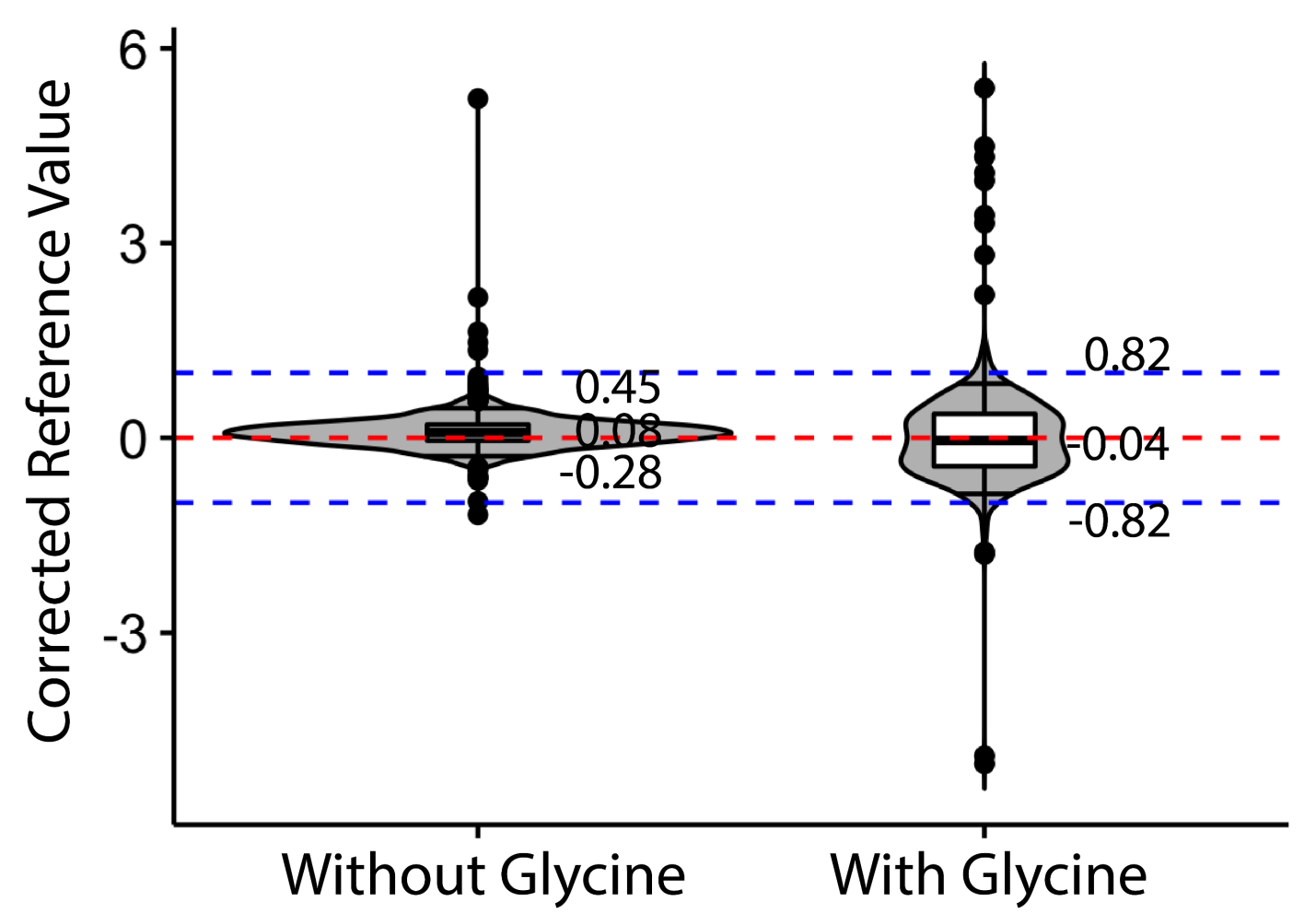
**

**Supplemental Figure 4. Performance of BaMORC methodology with and without glycine.** Inclusion of glycine-specific statistical models had a worse performance than leaving these statistical models out of the full BaMORC method. The violin plots here show the distribution of the results. The mark on the top of each plot is the 95% quantile and the one on the bottom is the 5% quantile. The boxplots show the 75%, 50% and 25% quantiles respectively.

**
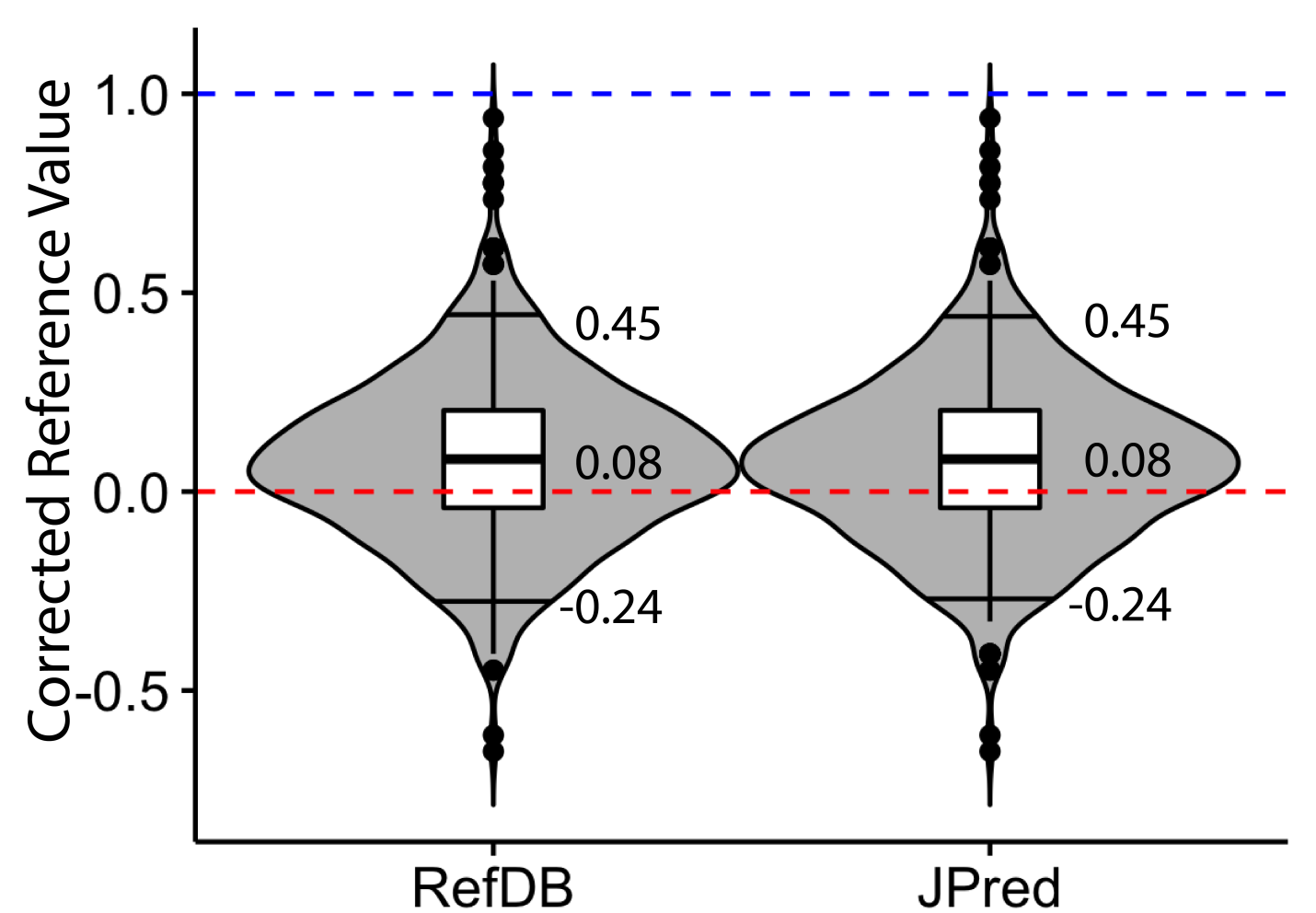
**

**Supplemental Figure 5. Comparison of the results obtained utilizing secondary structure information from RefDB and JPred4.** The BaMORC method performs equally well with secondary structure prediction from the JPred method as with the actual secondary structure information from the RefDB dataset. The violin plots here show the distribution of the results. The mark on the top of each plot is the 95% quantile and the one on the bottom is the 5% quantile. The boxplots show the 75%, 50% and 25% quantiles respectively.

**
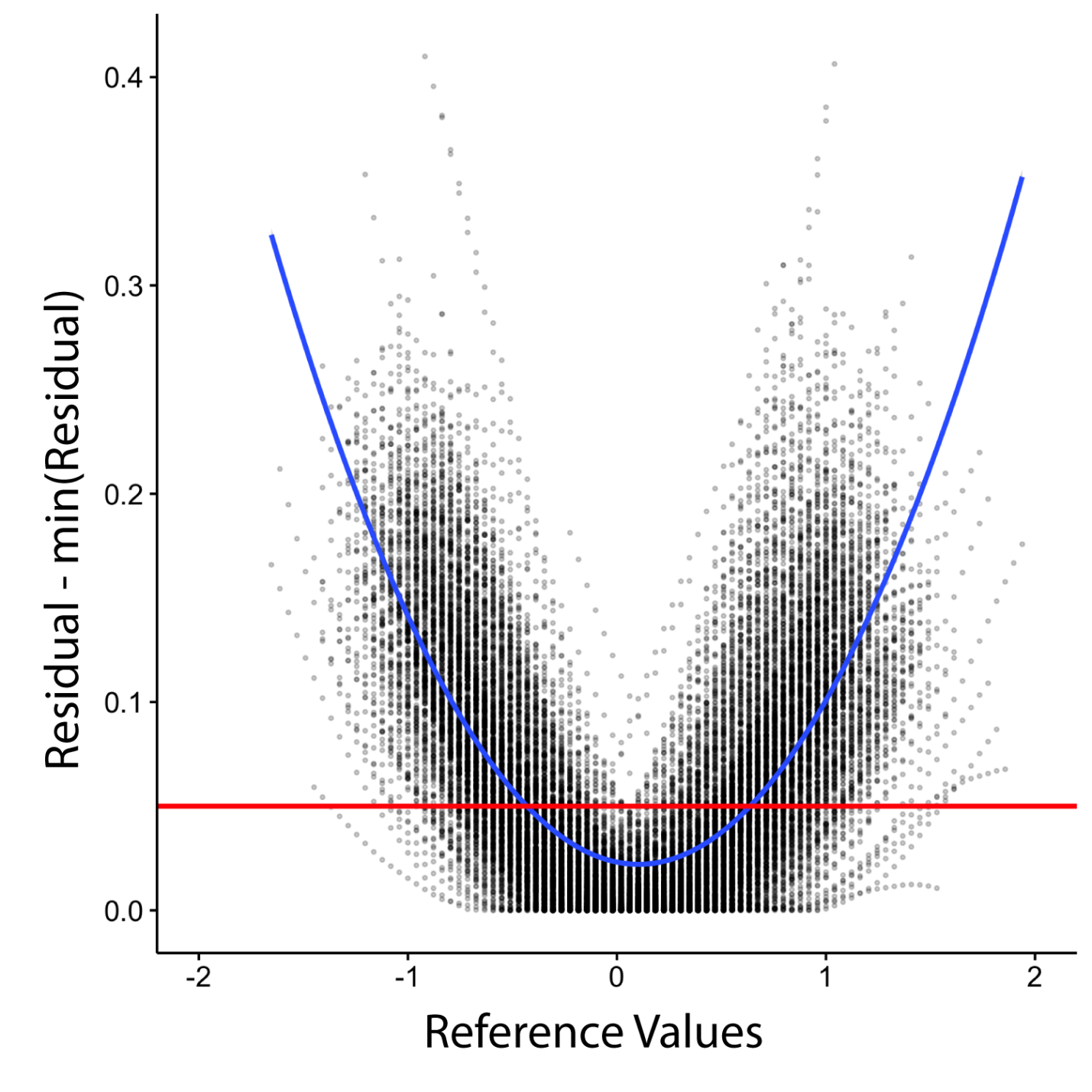
**

**Supplemental Figure 6. Amino Acid and Secondary Structure Frequency Residual of Residual vs. Reference Correction Values for RefDB datasets.** The y-axis is the residual of observed and predicted AA-SS frequencies from BaMORC minus the minimum residual observed corresponding to the Reference Correction Value on the x-axis. The blue line is the quadratic regression line to the values. The red line represents a 5% error rate above best amino acid and secondary structure prediction performance. The intersection of the red line with the blue line occurs at -0.43 ppm and 0.64 ppm.

**
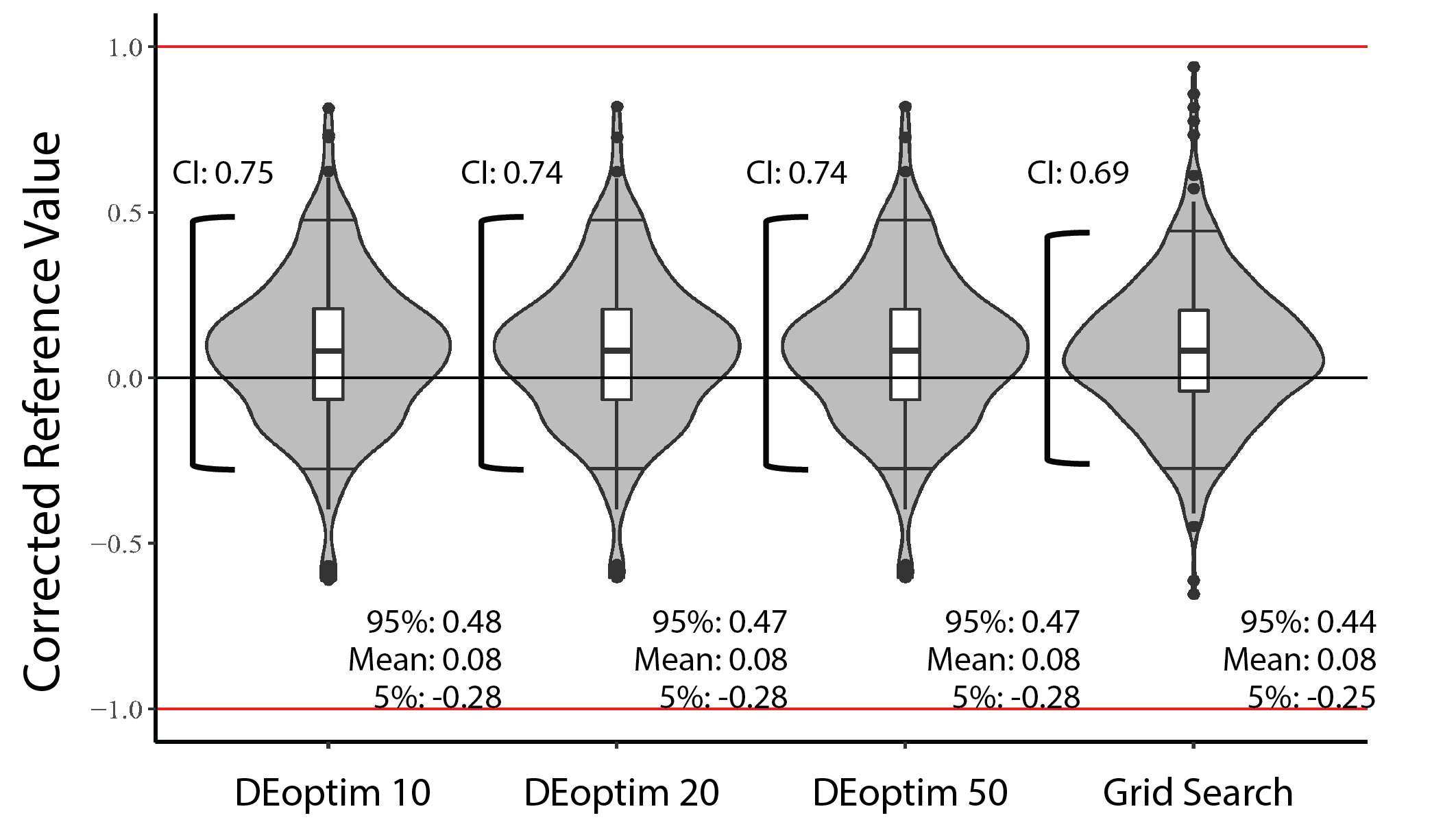
**

**Supplemental Figure 7. Comparison of BaMORC performance using grid search optimization vs global optimization by differential evolution.** To further reduce the computation time and allow a better user experience, we exchanged the grid-search approach with a function from Global Optimization by Differential Evolution (DEoptim) library (Mullen et al. 2009a). This global optimization function was implemented using the differential evolution algorithm (DE) (Price et al. 2006). Three max iteration parameters were used for DEoptim function: 10, 20, 50. The violin plots here show the distribution of the results. The mark on the top of each plot is the 95% quantile and the one on the bottom is the 5% quantile. The boxplots show the 75%, 50% and 25% quantiles respectively. The results from these three settings are very similar. With the higher iteration value, the results get better trivially. Round-up mean values are all 0.08 ppm, which is same the grid search algorithm. All of the DEoptim results have a 0.75 ppm range at the 90% confidence interval, which share the same trend of the mean values, and they are different from grid search by 0.05 ppm range at the 90% confidence interval.

**
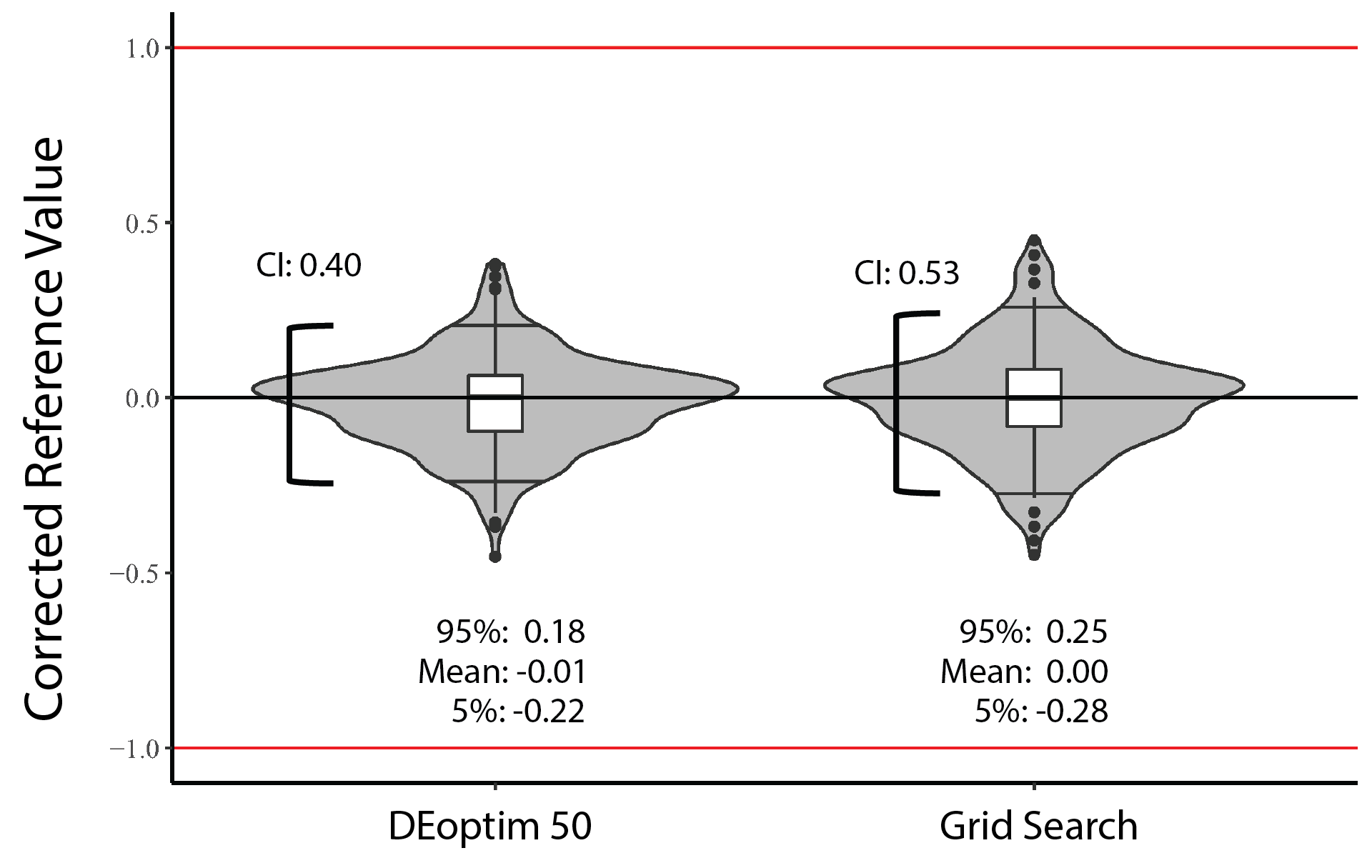
**

**Supplemental Figure 8. Comparison of Assigned BaMORC performance using grid search optimization vs global optimization by differential evolution.** The global optimization function was implemented using the differential evolution algorithm (DE) (Price et al. 2006). The results from DEoptim function with max iteration value equal to 50 performs much better than our original grid search implementation. The violin plots here show the distribution of the results. The mark on the top of each plot is the 95% quantile and the one on the bottom is the 5% quantile. The boxplots show the 75%, 50% and 25% quantiles respectively. The mean correction values are -0.01 and 0.00 respectively. The DEoptim results have a 0.40 ppm range at the 90% confidence interval, which share the significantly different from grid search by 0.13 ppm range at the 90% confidence interval.

**
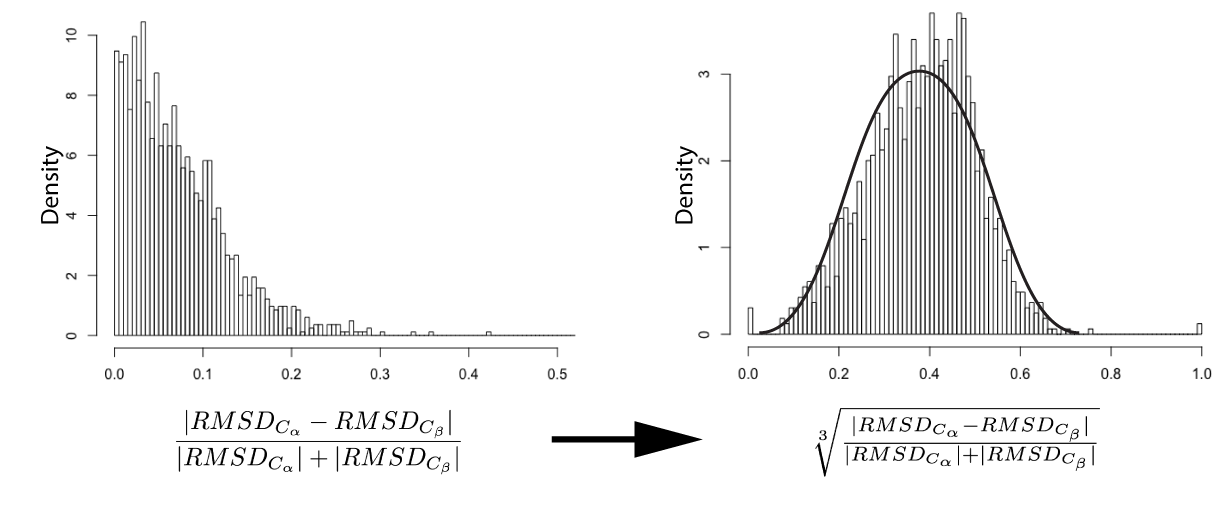
**

**Supplemental Figure 9. Cube root transformation**. On the left, the distribution of the resulting transformation is highly skewed, and it only approximates a normal distribution through a cube-root transformation.

**
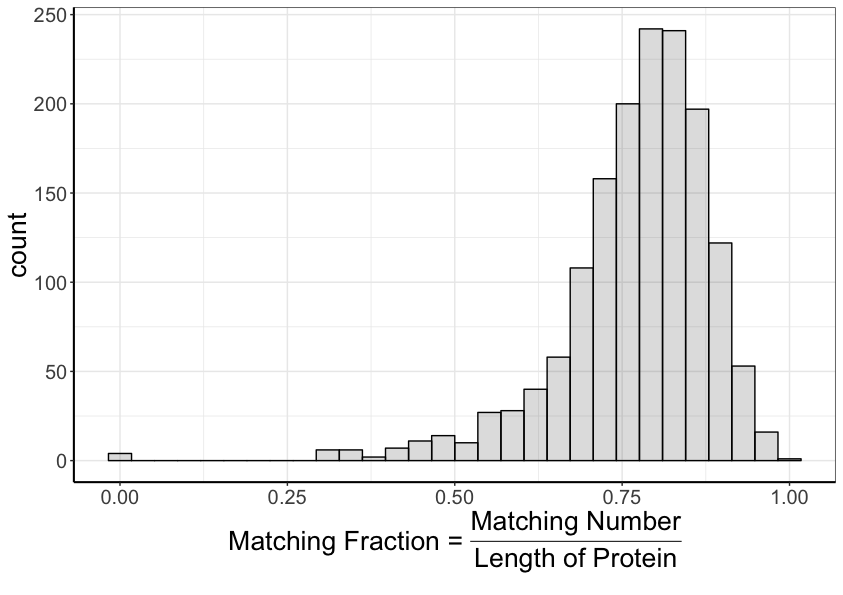
**

**Supplemental Figure 10. Performance (Matching Fraction) for JPred Algorithm on all RefDB datasets.** The histogram shows the resulting secondary structure estimation via JPred Algorithm with high performance. 1258 out of 1557 datasets have matching fraction over 0.7. 46718 correct out of 56015 total RefDB helix identified amino acids; 34063 correct out of 73048 total RefDB coil identified amino acids; 34063 correct out of 50930 total RefDB beta strand identified amino acids.

**Supplemental Tables**

|  | Ca Mean | | | Cb Mean | | | Ca SD | | | Cb SD | | | Covariance | | |
| --- | --- | --- | --- | --- | --- | --- | --- | --- | --- | --- | --- | --- | --- | --- | --- |
| Residue | Coil | Helix | Beta Strand | Coil | Helix | Beta Strand | Coil | Helix | Beta Strand | Coil | Helix | Beta Strand | Coil | Helix | Beta Strand |
| A | 52.84 | 54.83 | 51.53 | 19.06 | 18.26 | 21.14 | 1.64 | 1.05 | 1.48 | 1.26 | 0.88 | 2.05 | -0.58 | -0.31 | -0.99 |
| $C_{o}$ | 57.04 | 59.63 | 56.02 | 40.58 | 39.34 | 42.98 | 2.33 | 2.43 | 1.72 | 2.99 | 2.79 | 3.88 | 2.00 | 1.09 | 1.99 |
| $C_{r}$ | 57.51 | 61.58 | 56.57 | 29.50 | 27.47 | 30.08 | 2.49 | 2.89 | 1.76 | 1.97 | 1.37 | 1.69 | 3.00 | -0.37 | -0.40 |
| D | 54.18 | 56.70 | 53.87 | 40.85 | 40.51 | 42.30 | 1.60 | 1.61 | 1.64 | 1.32 | 1.33 | 1.62 | 4.00 | -0.48 | 0.05 |
| E | 56.87 | 59.11 | 55.50 | 30.20 | 29.37 | 32.01 | 1.82 | 1.16 | 1.67 | 1.55 | 0.99 | 1.98 | 5.00 | -1.00 | -0.18 |
| F | 57.98 | 60.81 | 56.65 | 39.45 | 38.78 | 41.54 | 2.02 | 1.90 | 1.59 | 1.98 | 1.31 | 1.74 | 6.00 | -0.22 | 0.32 |
| H | 55.86 | 59.04 | 55.09 | 29.97 | 29.54 | 31.85 | 1.96 | 1.74 | 1.78 | 2.42 | 1.46 | 2.22 | 7.00 | -0.17 | 0.28 |
| I | 61.03 | 64.57 | 60.05 | 38.65 | 37.60 | 39.86 | 1.90 | 1.74 | 1.57 | 1.69 | 1.15 | 1.98 | 8.00 | -0.72 | 0.44 |
| K | 56.59 | 58.93 | 55.40 | 32.79 | 32.27 | 34.63 | 1.78 | 1.44 | 1.34 | 1.67 | 0.88 | 1.78 | 9.00 | -0.82 | 0.02 |
| L | 54.92 | 57.52 | 54.00 | 42.38 | 41.65 | 43.79 | 1.70 | 1.23 | 1.31 | 1.64 | 1.05 | 2.00 | 10.00 | -0.54 | -0.31 |
| M | 55.67 | 58.09 | 54.58 | 33.36 | 32.27 | 35.05 | 1.54 | 1.81 | 1.24 | 2.26 | 1.66 | 2.29 | 11.00 | -0.75 | 1.13 |
| N | 53.23 | 55.45 | 52.74 | 38.55 | 38.61 | 40.12 | 1.51 | 1.42 | 1.47 | 1.41 | 1.31 | 2.07 | 12.00 | -0.46 | -0.20 |
| P | 63.47 | 65.49 | 62.64 | 31.94 | 31.46 | 32.27 | 1.26 | 1.08 | 1.03 | 0.95 | 0.95 | 1.20 | 13.00 | -0.05 | -0.20 |
| Q | 56.12 | 58.47 | 54.83 | 29.14 | 28.51 | 31.28 | 1.72 | 1.19 | 1.41 | 1.69 | 0.92 | 1.93 | 14.00 | -0.93 | -0.20 |
| R | 56.42 | 58.93 | 55.14 | 30.66 | 30.14 | 32.19 | 1.94 | 1.55 | 1.64 | 1.67 | 1.14 | 1.80 | 15.00 | -0.73 | 0.00 |
| S | 58.38 | 60.88 | 57.54 | 64.03 | 63.08 | 65.16 | 1.69 | 1.61 | 1.40 | 1.27 | 1.12 | 1.51 | 16.00 | -0.74 | -0.36 |
| T | 61.64 | 65.61 | 61.06 | 70.12 | 68.88 | 70.75 | 2.07 | 2.39 | 1.59 | 1.33 | 1.17 | 1.51 | 17.00 | -1.37 | -1.37 |
| V | 62.06 | 66.16 | 60.83 | 32.71 | 31.49 | 33.91 | 2.16 | 1.55 | 1.64 | 1.37 | 0.72 | 1.61 | 18.00 | -1.33 | -0.33 |
| W | 57.78 | 60.01 | 56.41 | 29.67 | 29.30 | 31.50 | 1.71 | 1.77 | 1.87 | 1.74 | 1.40 | 1.70 | 19.00 | -0.81 | -0.50 |
| Y | 57.97 | 60.98 | 56.83 | 38.95 | 38.25 | 40.97 | 2.17 | 1.76 | 1.71 | 1.84 | 1.11 | 1.85 | 20.00 | -0.12 | 0.20 |

**Supplemental Table 1.** The summary of alpha and beta ^13^C chemical shift statistics used in models explained in the manuscript.

| **Program** | **Detects or performs shift referencing** | **Detects gross assignment errors** | **Distinguishes assignment errors from referencing errors** | **Requires assigned chemical shifts** | **Requires 3D structure** |
| --- | --- | --- | --- | --- | --- |
| CheckShift (Wang et al. 2010b) | Yes | No | No | Yes | No |
| LACS (Wang et al. 2005) | Yes | No | No | Yes | No |
| PANAV (Wang et al. 2010b) | Yes | Yes | Yes | Yes | No |
| SHIFTX &  SHIFTCOR (Neal et al. 2003) | Yes | Yes | Yes | Yes | Yes |
| SPARTA+ (Yang and Bax 2010) | Yes | No | No | Yes | Yes |
| VASCO(Rieping and Vranken 2010) | Yes | Yes | Yes | Yes | Yes |
| AVS (Wang et al. 2005) | No | Yes | No | Yes | No |

**Supplemental Table 2.** Protein chemical shift re-referencing and assignment evaluation software.

| **Covariance** | **Estimated Reference Value (ppm)** |
| --- | --- |
| A | 0.327 |
| B | -0.0408 |
| C | 0.245 |
| D | -0.0408 |
| E | 0.327 |
| E-Revised | 0.000 |

**Supplemental Table 3.** Performance of different covariance matrices on the BMR6032 dataset.

| **Covariance** | **5% (ppm)** | **25% (ppm)** | **50% (ppm)** | **75% (ppm)** | **95% (ppm)** | **90% IQR** | **50% IQR** |
| --- | --- | --- | --- | --- | --- | --- | --- |
| A | -0.24 | 0.16 | 0.53 | 1.18 | 2.33 | 2.57 | 1.02 |
| B | -0.33 | 0.33 | 0.82 | 1.30 | 2.12 | 2.94 | 0.97 |
| C | -0.24 | 0.08 | 0.32 | 0.73 | 2.13 | 2.37 | 0.65 |
| D | -0.37 | 0.00 | 0.28 | 0.57 | 1.43 | 1.80 | 0.57 |
| E | -0.41 | 0.33 | 0.73 | 1.14 | 1.79 | 2.20 | 0.81 |
| E-Revised | -0.82 | -0.41 | -0.20 | 0.04 | 0.53 | 1.35 | 0.45 |
| E-Revised + Overlap Matrix | -0.28 | -0.04 | 0.08 | 0.20 | 0.45 | 0.73 | 0.24 |
| E-Revised + Overlap Matrix (90% Completion) | -0.24 | -0.04 | 0.08 | 0.20 | 0.45 | 0.69 | 0.24 |

**Supplemental Table 4.** Quantiles and IQRs results for a series of statistical models tested against all of the data from the RefDB.

| **Completeness** | **5% (ppm)** | **25% (ppm)** | **50% (ppm)** | **75% (ppm)** | **95% (ppm)** | **90% IQR** | **50% IQR** |
| --- | --- | --- | --- | --- | --- | --- | --- |
| 100% | -0.24 | -0.04 | 0.08 | 0.20 | 0.45 | 0.69 | 0.24 |
| 95% | -0.29 | -0.08 | 0.08 | 0.20 | 0.45 | 0.69 | 0.24 |
| 90% | -0.24 | -0.08 | 0.08 | 0.20 | 0.43 | 0.67 | 0.24 |
| 85% | -0.29 | -0.08 | 0.08 | 0.21 | 0.45 | 0.69 | 0.26 |
| 80% | -0.29 | -0.08 | 0.04 | 0.24 | 0.49 | 0.73 | 0.29 |
| 75% | -0.30 | -0.08 | 0.08 | 0.24 | 0.49 | 0.73 | 0.29 |
| 70% | -0.33 | -0.08 | 0.08 | 0.24 | 0.53 | 0.78 | 0.29 |
| 65% | -0.33 | -0.12 | 0.08 | 0.21 | 0.49 | 0.73 | 0.26 |
| 60% | -0.41 | -0.12 | 0.08 | 0.24 | 0.57 | 0.82 | 0.29 |
| 55% | -0.37 | -0.12 | 0.08 | 0.24 | 0.53 | 0.78 | 0.29 |
| 50% | -0.41 | -0.12 | 0.08 | 0.24 | 0.57 | 0.82 | 0.29 |

**Supplemental Table 5.** Quantiles and IQRs for the robustness testing of the BaMORC method.

| **Secondary Structure** | **5%**  **(ppm)** | **25% (ppm)** | **50% (ppm)** | **75% (ppm)** | **90% (ppm)** | **90% IQR (ppm)** | **50% IQR (ppm)** |
| --- | --- | --- | --- | --- | --- | --- | --- |
| RefDB | -0.24 | -0.4 | 0.08 | 0.20 | 0.45 | 0.69 | 0.24 |
| JPred | -0.24 | -0.04 | 0.08 | 0.20 | 0.45 | 0.69 | 0.24 |

**Supplemental Table 6.** Quantiles and IQRs from the results of the BaMORC method performed using secondary structure information from RefDB and JPred.
